# SILAC-iPAC: A quantitative method for distinguishing genuine from non-specific components of protein complexes by parallel affinity capture

**DOI:** 10.1016/j.jprot.2014.12.006

**Published:** 2015-02-06

**Authors:** Johanna S. Rees, Kathryn S. Lilley, Antony P. Jackson

**Affiliations:** a Department of Biochemistry, University of Cambridge, Cambridge CB2 1QW, UK; b Cambridge Centre for Proteomics, University of Cambridge, Cambridge CB2 1QR, UK

**Keywords:** AP-MS, Affinity purification mass spectrometry, CS, Chicken serum, FA, Fanconi anaemia, FDR, False discovery rate, LTQ, Linear trap quadrupole, PI5P4K2β/α, Phosphatidyl inositol 5-phosphate 4-kinase 2 β/α subunits, PPI, Protein protein interactions, SILAC–iPAC, Stable isotope labelling of amino acids in culture-interactomes by parallel affinity capture, TAP, Tandem-affinity purification, UPLC, Ultra performance liquid chromatography., SILAC–iPAC, Protein–protein interactions, Beadomes, Phosphatidyl-inositol-5-phosphate-4-kinase-2β, Fanconi anaemia

## Abstract

Pull-down assays can identify members of protein complexes but suffer from co-isolation of contaminants. The problem is particularly acute when the specifically interacting partners are of low-abundance and/or bind transiently with low affinity. To differentiate true interacting partners from contaminants, we have combined SILAC labelling with a proteomic method called “Interactomes by Parallel Affinity Capture” (iPAC). In our method, a cell-line stably expressing a doubly tagged target endogenous protein and its tag-less control cell-line are differentially SILAC labelled. Lysates from the two cell-lines are mixed and the tagged protein is independently purified for MS analysis using multiple affinity resins in parallel. This allows the quantitative identification of tagged proteins and their binding partners. SILAC–iPAC provides a rigorous and sensitive approach that can discriminate between genuine binding partners and contaminants, even when the contaminants in the pull-down are in large excess. We employed our method to examine the interacting partners of phosphatidyl inositol 5-phosphate 4-kinase 2β subunit (PI5P4K2β) and the Fanconi anaemia core complex in the chicken pre-B cell-line DT40. We confirmed known components of these two complexes, and we have identified new potential binding partners. Combining the iPAC approach with SILAC labelling provides a sensitive and fully quantitative method for the discrimination of specific interactions under conditions where low signal to noise ratios are unavoidable. In addition, our work provides the first characterisation of the most abundant proteins within the DT40 proteome and the non-specific DT40 ‘beadomes’ (non-specific proteins binding to beads) for common epitope tags. Given the importance and widespread use of the DT40 cell-line, these will be important resources for the cell biology and immunology communities.

Biological significance

SILAC–iPAC provides an improved method for the analysis of low-affinity and/or low abundance protein-protein interactions. We use it to clarify two examples where the nature of the protein complexes are known, or are currently unclear. The method is simple and quantitative and will be applicable to many problems in cell and molecular biology. We also report the first chicken beadomes.

## Highlights

• SILAC–iPAC; an improved AP-MS method to quantitatively detect low abundance proteins
• RUVBL1 and its partner RUVBL2 are novel interactors of the Fanconi anaemia complex.
• First characterisation of chicken DT40 beadomes using four common epitope tags

## Graphical abstract

**Figure:**
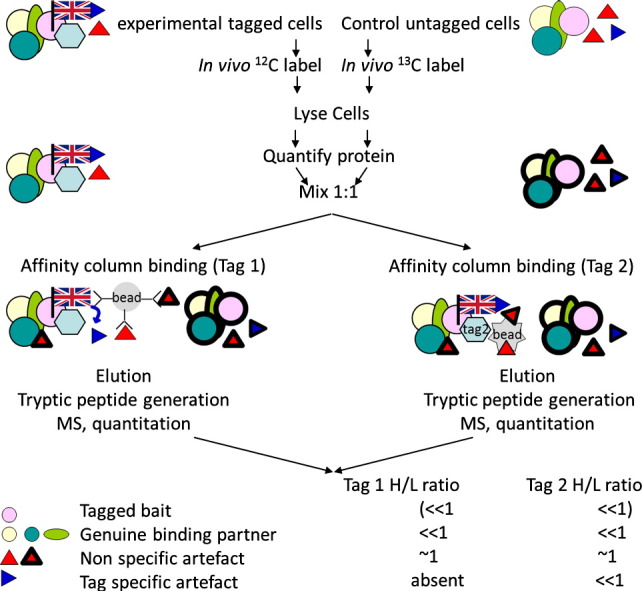

## 1 Introduction

Proteins often functionally interact as part of complex and dynamic networks [1,2]. An important goal of current proteomics research is to develop techniques that can accurately identify such protein ‘interactomes’ [3]. A popular method uses epitope-tagged proteins in pull-down assays. For example, tandem-affinity purification (TAP-tagging) methodology uses a ‘bait’ protein containing two separate epitope tags in series, separated by a protease-cleavable site. This enables sequential purification, with cleavage at the protease site used to bring about elution from the first affinity matrix column [4]. In principle, the serial nature of the purification should provide higher stringency and lower false positive rates compared to pull-downs of single tagged proteins. Unfortunately, this serial approach often compromises recovery, making yield a limiting factor and selecting for only the most stable complexes. Furthermore, the extended time course inevitably associated with TAP-tagging may lead to proteins being more vulnerable to proteolytic degradation [2,5]. These technical limitations are becoming more apparent as experimentalists increasingly examine the functional interactions of low abundance proteins, or proteins that bind transiently and with low affinity. Although cross-linking can be used to stabilise transient interactors, the shortcomings are that non-specific interactors would also cross-link, and perhaps in different proportions as the controls. Many proteins that play critical roles in cell signalling, DNA replication and other key pathways fall into these categories. Hence there is a clear need for improved methods of proteomic analysis that can be applied in such cases.

The assignment of proteins as genuine ‘bait’ partners is often qualitative. But as instrument sensitivity improves, it is becoming increasingly difficult to discriminate between specific and non-specific proteins from pull-downs by purely qualitative means or by simple presence/absence criteria [6]. To provide quantitation, label free methods such as spectral counting using SAINT or emPAI scoring using APEX have been adopted [7]. As an alternative, metabolic labelling of samples as early as possible offers significant improvement in precision, because it can minimise technical variability [8]. A more fundamental problem is that pull-down experiments typically generate long lists of proteins, most of which are likely to be non-specific contaminants [2,9]. Moreover, different affinity matrix resins can bind different subsets of proteins non-specifically and identifying contaminants that are selectively associated with particular resins is a problem that has only recently begun to be appreciated and addressed [2,10].

We have previously described the ‘interactomes by parallel affinity capture’ (iPAC) method [2,5]. Here, a doubly-tagged protein is affinity purified using each tag in separate parallel experiments. Genuine binding partners are identified by their consistent presence in both independent pull-downs [2,5]. We now combine iPAC with stable isotope labelling of amino acids in cell culture (SILAC) [11], to provide a fully quantitative characterisation of specific and non-specifically-bound proteins (SILAC–iPAC). Whilst AP-MS coupled with SILAC has been performed successfully [12], SILAC–iPAC offers additional precision with its combination of quantitative proteomics and multiple tag approaches, and is particularly designed for low-abundance interactors. For our proof of concept experiments, we have chosen two challenging examples where the target proteins are present in cells at only low levels, and where previous proteomic analysis has been limited or ambiguous. These are the β-subunit of phosphatidyl inositol 5-phosphate 4-kinase (PI5P4K2β) [13], and the Fanconi anaemia core complex member FANCC [14], both in the chicken pre-B cell-line DT40.

The DT40 cell-line exhibits an extraordinarily high rate of homologous recombination. It is therefore possible to generate cell-lines containing specific gene targeted constructs. Since these constructs are regulated by their natural promoters, the epitope-tagged proteins are expressed at their physiologically normal levels [15]. The DT40 cell line JPR3 contains one PI5P4K2β allele modified to encode a tandem C-terminal 3 × FLAG tag and two (6 × His) tags [13]. PI5P4K2β can thus be isolated using immobilised anti-(FLAG epitope) or metal affinity resin. The enzyme PI5P4K2β is localised in the nuclear membrane, and co-assembles with its partner PI5P4K2α [16]. The enzyme catalyses the 4 phosphorylation of PtdIns5 to generate Ptdins (4,5) P2, a lipid regulator of proteins. Based on semi-quantitative affinity purifications it is likely that there is less than 1.5 pmole of PI5P4K2α per mg of total cell extract. This corresponds to about 9 × 10^4^ molecules of enzyme per cell, and the level of PI5P4K2β is probably at least 5 fold lower [16,17]. As well as the presence of homodimers, these authors report a new heterodimeric PI5P4K2α: PI5P4K2β association and no other significant interacting proteins as it is likely that binding of this enzyme to the nuclear membrane occurs solely *via* lipid interactions. We therefore use this established interaction as a positive control to test our SILAC–iPAC approach.

The Fanconi anaemia protein FANCC is a component of a multi-protein core complex that promotes homologous recombination and mutational repair of DNA interstrand crosslinks. Mutational disruption of this complex is responsible for the human chromosome breakage syndrome Fanconi anaemia (FA) [18]. The FANC complex typically exists as a low-abundance complex in growing cells. In the DT40 FANCC cell-line, the FANCC allele is modified to encode a C-terminal tandem calmodulin binding protein (CBP) and a Protein-A tag [14]. FANCC can thus be isolated using immobilised Calmodulin or IgG resin.

In both of these examples, proteomic analysis represents a significant technical challenge and provides experimental opportunities to test the potential of SILAC–iPAC. Here we demonstrate that by combining stable isotope labelling with the iPAC protocol we can distinguish between true interacting partners from co-isolating contaminants.

## 2 Materials and methods

### 2.1 Cell lines, cell maintenance and harvesting

Wild type DT40, JPR3 and FANCC cells were all grown in RPMI media with L-glutamine supplemented with 10% FBS and 1% chicken serum (CS) (all GIBCO) and maintained at 37 °C at 5% CO_2,_ as described previously [19]. Cells were counted using an C6 Accuri flow cytometer and lysed in lysis buffer comprising 1 × PBS (pH7.4) 1% Triton X-100, 1 mM PMSF and 1.5 × EDTA free protease inhibitor cocktail (Roche).

### 2.2 SILAC labelling

Exponential cells were transferred to SILAC K6R6 or K0R0 RPMI media (Dundee Cell Products, UK) containing 10% dialysed FBS and 1% dialysed CS. Incorporation of ^13^C was measured by LCMS of crude lysates over 10 days and found to be approximately 98% by day 8 based on spectra from 5 proteins differing in abundance, turnover, cellular region including the tagged bait.

### 2.3 Affinity purifications

Initial studies to identify abundant DT40 proteins, three replicates of 10^6^ total cells were lysed in 10 ml lysis buffer on ice and the cleared supernatants separated by reducing SDS-PAGE and stained. All bands were excised and the sample prepared and analysed by MS as described in ‘Sample preparation for MS/MS’ and ‘Mass spectrometry’ below. Data was processed using Mascot (Matrix Science) and xml outputs processed using ProteinCenter Version 3.13.2003 (Thermo).

**Fig. 1:**
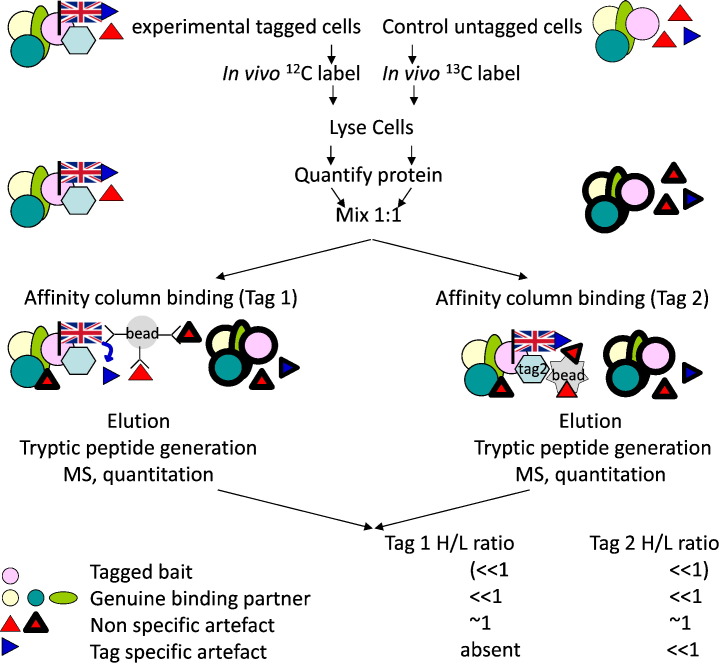
SILAC–iPAC experimental outline using tagged PI5P4K2βas an example.

For DT40 beadomes and SILAC–iPAC experiments all cell lines were grown in parallel to 10^8^ total cells maintained at 10^6^ cells/ml. Cells were lysed in 10 ml lysis buffer on ice and the cleared supernatants quantified, then added to 100 μl prewashed resins. For SILAC–iPAC experiments quantified lysates were mixed 1:1 (typically 5 mg) then added to 100 μl prewashed resins. JPR3 and wt DT40 cell extracts were purified using EZview ANTI- FLAG M2 Affinity Gel (Sigma) and TALON Metal Affinity Resin (Clontech). FANCC and wild-type DT40 cell extracts were purified with Calmodulin Sepharose 4B and IgG Sepharose 6 (both GE Healthcare). Native protein complexes were allowed to bind for 1 h then non-bound proteins removed by centrifugation at 2000 ×*g*, the resins washed 3 times for 15 minutes in 1 ml lysis buffer and the bound proteins eluted in 100 μl of elution buffer. Elution buffers were lysis buffer pH 7.4 containing either 100 μg/ml 3 × FLAG peptide for FLAG resin, 150 mM imidazole for TALON resins, or 200 mM EDTA for calmodulin resins. 100 mM glycine pH 2.5 was used for IgG resins. The experimental outline is depicted in Fig. 1*A*. Western blots were also performed using anti-tag antibodies (Sigma) or monoclonal antibodies to FANCC, FANC-G and FANC-M (all gifts from E. Rajendra).

### 2.4 Sample preparation for MS/MS

Eluates were acetone precipitated overnight at − 80 °C, resuspended in LDS sample buffer and resolved for ~ 2 cm on 10% reducing SDS-PAGE gels (Invitrogen). Analysis gels were Coomassie stained and four equal sized portions of the stained area were excised, washed, reduced in 2 mM DTT for 1 h at RT, alkylated in 10 mM iodoacetamide for 30 min at RT, and in-gel digested with 2 μg sequencing-grade porcine trypsin (Promega) overnight at 37 °C. Digests were concentrated using a speedvac and resuspended in 20 μl 0.1% formic acid.

### 2.5 Mass spectrometry

All LC-MS/MS experiments were performed using a nanoAcquity UPLC (Waters Corp., Milford, MA) system coupled to an LTQ Orbitrap Velos hybrid ion trap mass spectrometer (Thermo Scientific, Waltham, MA). Separation of peptides was performed by reverse-phase chromatography using a Waters reverse-phase nano column (BEH C18, 75 μm i.d. × 250 mm, 1.7 μm particle size) at flow rate of 300 nl/min. 10 μl peptide sample solution was initially loaded onto a pre-column (Waters UPLC Trap Symmetry C18, 180 μm i.d. × 20 mm, 5 μm particle size) from the nanoAcquity sample manager with 0.1% formic acid for 3 min at a flow rate of 10 μl/min. After this period, the column valve was switched to allow the elution of peptides from the pre-column onto the analytical column. Solvent A was water + 0.1% formic acid and solvent B was acetonitrile + 0.1% formic acid. The linear gradient employed was 5–35% B in 60 min. Washes with 0.1% formic acid were performed in between each biological sample type to minimise carryover. The Velos was operated in data-dependent mode with a dynamic exclusion of 0.3 Da *m/z*.

The LC eluant was sprayed into the mass spectrometer by means of a nanospray source (Thermo). All *m/z* values of eluting ions were measured in the Orbitrap Velos mass analyser, set at a resolution of 30,000. Data dependent scans (Top 10) were employed to automatically isolate and generate fragment ions by collision-induced dissociation in the linear ion trap, resulting in the generation of MS/MS spectra. Ions with charge states of 2 + and above were selected for fragmentation.

### 2.6 Protein identification

Post-run, the data was processed using Protein Discoverer (version 1.2., ThermoFisher). Briefly, all MS/MS data were converted to .mgf files and these files were then submitted to the Mascot search algorithm (v 2.3, Matrix Science, London UK) and searched against the UniprotKB *Gallus gallus* database (31,529 protein entries, 2012), using a fixed modification of carbamidomethyl (C) and a variable modification of oxidation (M), allowing 2 missed cleavages, a peptide mass tolerance of 25 ppm and a fragment mass tolerance of 0.8 Da. Significance threshold was set to *P* > 0.01 and ion score cut off was set to 20. FDR was calculated using the reverse *Gallus* database and only proteins with an FDR of < 5% were considered. In addition Mascot Percolator was used for scoring and to compare protein lists. For quantitation we used MaxQuant v 1.3 [20]. Default parameters were used with FDR of 0.01%, and a minimum of two unique and razor peptides were selected for quantifying proteins. We used the MaxQuant generated normalised data for quantitative comparison across replicates and reciprocal labelling experiments and used Microsoft excel to plot the Log_2_ ratio distributions. We set a significance threshold of a ratio > +/− 1 SD of the median of the entire normalised data set. Data processing and filtering are outlined in supplemental Fig. S1. Venn diagrams were produced using http://bioinfogp.cnb.csic.es/tools/venny/index.html [21]*.*We used STRING v 1.9 to search for known interactors with emphasis on experimental evidence [22].

## 3 Results

### 3.1 SILAC–iPAC

The SILAC–iPAC schema is summarised in Fig. 1. Here a ‘bait’ protein contains two different epitope tags (1 and 2) in series. However, unlike traditional TAP-tagging, there is no need to separate the epitope tags with a protease-cleavable site. Rather, a cell line expressing the double-tagged protein is grown in light (L) SILAC media, and control cells lacking tagged protein are grown in heavy (H) SILAC media. Cells are lysed, and equal amounts of proteins from the two samples mixed together. In parallel experiments, the mixed lysates are separately incubated with anti-(tag 1) and anti-(tag 2) resins. Proteins are eluted from the columns, peptides are prepared, then analysed by standard LC-MS. Peptides corresponding to individual proteins are identified and their H/L ratios are quantified. The experiment is repeated but with reciprocal labelling, such that cells expressing doubly-tagged bait are grown in heavy SILAC media and control cells in light SILAC media. The ‘bait’ protein and its genuine binding partners will exhibit isotope ratios that deviate significantly from 1 in both parallel affinity purifications.

In a clean pull-down, a highly specific interaction between tag and resin will yield the tagged protein with the isotope signature corresponding to the sample labelling with no or few corresponding peptides from the control cells. In such a case, the H/L ratios cannot be reported. However with the increased sensitivity of MS instrumentation, complete presence/absence is rarely seen. In contrast, any non-specific matrix binding proteins will appear in both samples, since these proteins are expected to bind the matrix equally from tagged and untagged cells. These proteins will display H/L ratios close to 1. High abundance proteins that specifically bind to the bait may be present with isotopic peptides corresponding to both tagged and control cells. However, these proteins will display H/L ratios that deviate significantly from 1 (Fig. 1). Thus, SILAC quantitation will discriminate the genuine from non-specific binders.

It is possible that different affinity resins could potentially bind distinct patterns of non-specific proteins. However, the peptides from such proteins will still exhibit H/L isotope ratios of ~ 1, although in this case the proteins will be more prominently or exclusively associated with individual affinity resins. In addition, proteins that bind to the tag itself could interfere with specific binding by reducing association with the resin. Alternatively, where a low affinity non-specific protein binds the tag, a higher affinity resin-binding competitor peptide will compete off the tagged protein for that corresponding resin but not for the alternate resin, giving no ratio for the first case and a deviation of 1 in the alternate resin (depicted by the ‘tag specific artefact’ in Fig. 1). An outline of the data processing workflow and acceptance criteria is depicted in supplemental Fig. S1 (Fig. 3 in [23]).

### 3.2 The DT40 ‘beadome’: Interaction of DT40 proteins with different affinity resins

In preliminary experiments to gauge the abundant proteins in a crude lysate and the composition of non-specific binders, we performed an MS analysis on proteins from replicate DT40 cell lysates, and identified those that were captured by each of the four different affinity resins: FLAG, TALON, IgG and calmodulin sepharose. We report the top 150 abundant proteins as ranked by emPAI score (supplemental Table S1*A*, Table 1 in [23]). A GO annotation was performed and identified 53 over-represented proteins that included ribosomes, viral proteins, vesicular and mitochondrial proteins. The under-represented proteins included membrane and extracellular matrix proteins (supplemental Fig. S2, Fig. 1 in [23]). Neither our cytoplasmic bait proteins PI5P4K2β and FANCC, nor any other cytoplasmic or nuclear FANC proteins were present in this list, confirming their lower abundance.

In preliminary experiments to gauge the abundant proteins in a crude lysate and the composition of non-specific binders, we performed an MS analysis on proteins from replicate DT40 cell lysates, and identified those that were captured by each of the four different affinity resins: FLAG, TALON, IgG and calmodulin sepharose. We report the top 150 abundant proteins as ranked by emPAI score (supplemental Table S1*A*, Table 1 in [23]). A GO annotation was performed and identified 53 over-represented proteins that included ribosomes, viral proteins, vesicular and mitochondrial proteins. The under-represented proteins included membrane and extracellular matrix proteins (supplemental Fig. S2, Fig. 1 in [23]). Neither our cytoplasmic bait proteins PI5P4K2β and FANCC, nor any other cytoplasmic or nuclear FANC proteins were present in this list, confirming their lower abundance.

The DT40 beadomes identified a total of 367 proteins (1.16% of the *Gallus* proteome) of which 150 (41%) bound to two or more different affinity resins (supplemental Table S1*B*, Table 2 in [23]). These proteins were predominantly cytoplasmic and ribosomal as classified by Gene Ontology and Kegg pathway annotations (supplemental Fig. S3, Fig. 2 in [23]). Almost half (45%) of these proteins were present in the top 150 abundant DT40 protein list highlighting the fact that abundant proteins can non-specifically bind any affinity resin and mask genuine interactors.

The DT40 beadomes identified a total of 367 proteins (1.16% of the *Gallus* proteome) of which 150 (41%) bound to two or more different affinity resins (supplemental Table S1*B*, Table 2 in [23]). These proteins were predominantly cytoplasmic and ribosomal as classified by Gene Ontology and Kegg pathway annotations (supplemental Fig. S3, Fig. 2 in [23]). Almost half (45%) of these proteins were present in the top 150 abundant DT40 protein list highlighting the fact that abundant proteins can non-specifically bind any affinity resin and mask genuine interactors.

These initial studies provide data on the nature of potential contaminants, and further highlight the problem of discriminating specific interactors from non-specific proteins in any type of affinity purification of native complexes.

### 3.3 PI5P4K2β

**Fig. 2:**
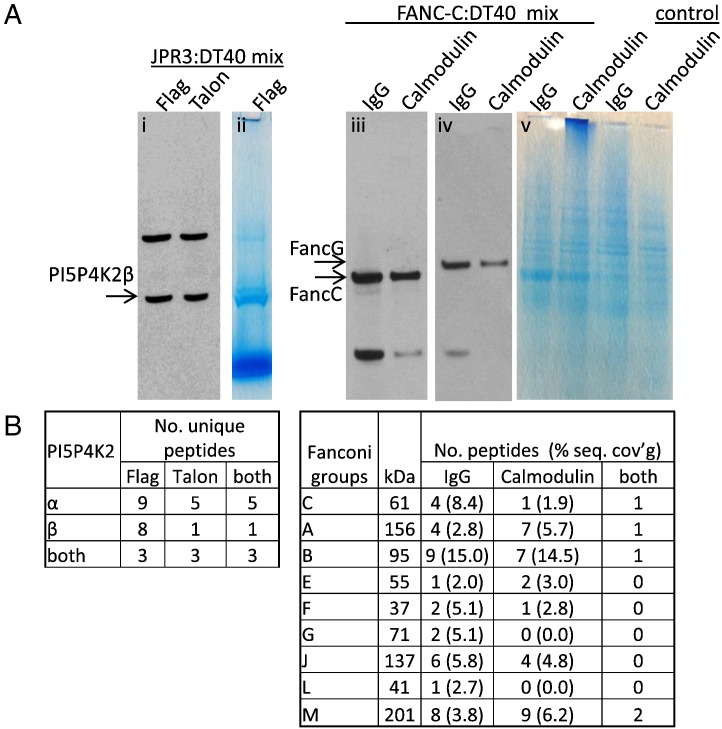
*(A)* Identification of the bait proteinsPI5P4K2β (JPR3 cell line) using Flag MAb (i) and coomassie (ii) and FANCC using αProtein A (iii) and rabbit αFANCG (iv) and Coomassie (v) in parallel pulldowns compared to control DT40 eluates (two right hand lanes). *(B)* PI5P4K2peptides and *(C)* Fanconi peptides identified by mass spectrometry. (see Supplemental Tables S2A and B for all peptide sequences). *(A)* Identification of the bait proteinsPI5P4K2β (JPR3 cell line) using FLAG MAb (i) and coomassie (ii) and FANCC using αProtein A (iii) and rabbit αFANCG (iv) and coomassie (v) in parallel pull-downs compared to control DT40 eluates (two right hand lanes). *(B)* PI5P4K2 peptides and *(C)* Fanconi peptides identified by mass spectrometry. (see Supplemental Tables S2A and B for all peptide sequences).

To test our SILAC–iPAC method we performed the procedure on the known phosphatidyl inositol 5-phosphate 4-kinase 2 heterodimeric complex comprising PI5P4K2α and PI5P4K2β. Previous qualitative and semi-quantitative MS analysis of this low-abundance enzyme identified both subunits, but could not resolve the issue of other potential interactors [16]. Four biological replicates were performed for the PI5P4K2β as well as reciprocal labellings for each, totalling 8 replicates per affinity pull-down. We were able to detect both PI5P4K2β and its known partner PI5P4K2α with sequence coverages of 35% and 38% respectively for Flag pull-downs. TALON pulldowns generated fewer peptides, with maximum of 9% and 25% sequence coverages respectively, most of which were reproducible with FLAG pull-downs demonstrating the utility and importance of parallel affinity purifications. The presence of bait protein and resulting peptides by MS is shown in Fig. 2, supplemental Fig. S4 and supplemental Table S2*A.*

To test our SILAC–iPAC method we performed the procedure on the known phosphatidyl inositol 5-phosphate 4-kinase 2 heterodimeric complex comprising PI5P4K2α and PI5P4K2β. Previous qualitative and semi-quantitative MS analysis of this low-abundance enzyme identified both subunits, but could not resolve the issue of other potential interactors [16]. Four biological replicates were performed for the PI5P4K2β as well as reciprocal labellings for each, totalling 8 replicates per affinity pull-down. We were able to detect both PI5P4K2β and its known partner PI5P4K2α with sequence coverages of 35% and 38% respectively for FLAG pull-downs. TALON pull-downs generated fewer peptides, with maximum of 9% and 25% sequence coverages respectively, most of which were reproducible with FLAG pull-downs demonstrating the utility and importance of parallel affinity purifications. The presence of bait protein and resulting peptides by MS is shown in Fig. 2, supplemental Fig. S4 and supplemental Table S2*A.*

**Fig. 3:**
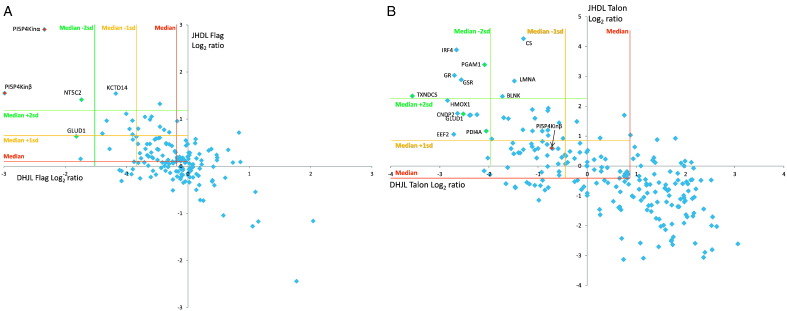

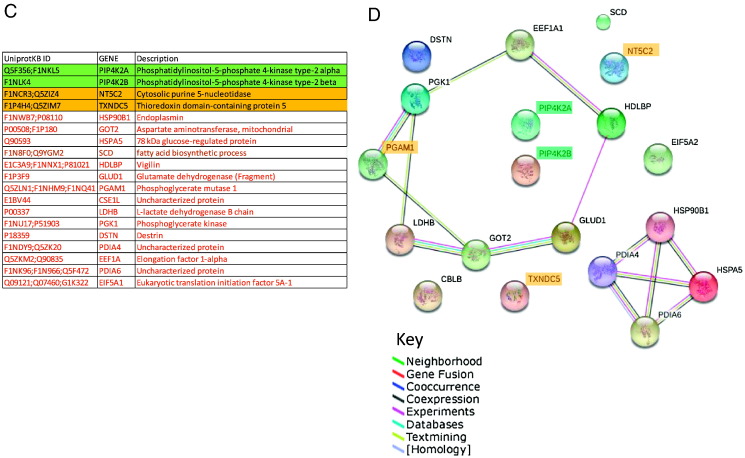
(*A*&*B*) Scatter plots to show the correlation of Log_2_ protein ratios from a representative replicate for the PI5P4K2β pull-downs with control DT40 Heavy SILAC labelling, JPR3 Light labelling (DHJL) and the reciprocal labelling experiments (JHDL) respectively for FLAG *(A)* and TALON *(B). (C)* List of significant (> 1SD of median) proteins from FLAG and TALON pull-downs of 4 combined replicates with green, orange and red being high, medium and low confidence respectively. *(D)* STRING interaction map of the significant candidates. (See Supplemental Tables S4 for full list of identified proteins and ratios.)

Consistent with its low abundance, we could not quantify PI5P4K2β in the first two replicates. However upon increasing cell numbers, we were able to quantify both isoforms. We therefore used datasets 3 and 4 for mining interactors. We found good correlation of SILAC ratios when comparing the replicates and reciprocals (Fig. 3A, B) and for FLAG pull-downs both PI5P4K2β and PI5P4K2α had statistically significant ratios (> 2 SD from the median) as well as one other protein NT5C2, a cytosolic purine 5-nucleotidase.The TALON data showed alternate proteins that were of greatest significance with the PI5P4K2 being only 1SD from the median ratio. Of the 218 FLAG and 348 TALON purified proteins 84 proteins were identified using the two different affinity tags. We took all proteins with > 1 SD of median from each of the four FLAG and TALON datasets (Supplemental Table 3A&B, Table 3 in [23]) and found that only 18 were in common for both pulldowns. These are listed in Fig. 3C and STRING interaction mapping was performed to analyse the validity of these (Fig. 3D). STRING clearly showed both the PI5P4K2β and PI5P4K2α isoforms as well as NT5C2, TXNDC5 and SCD as non-interaction proteins distinct from a large cluster of mostly metabolic related proteins and a smaller cluster of protein processing proteins (PDIAs and chaperones). To check the validity of these non-interacting proteins we expanded the string network to include increments of ten additional known interactors (Supplemental Fig. S6) and still did not find a connecting protein for PI5P4K2β and PI5P4K2α thus concluding that these isoforms do not interact with any other proteins. For proteins NT5C2, TXNDC5 and SCD we have ranked these as high, medium or low confidence based on the identification and ratio reproducibility amongst replicated, reciprocal and tags (Fig. 3D). NT5C2, a cytosolic protein may have a critical role in the maintenance of a constant composition of intracellular purine/pyrimidine nucleotides in cooperation with other nucleotidases, and preferentially hydrolyses inosine 5′-monophosphate (IMP) and other purine nucleotides. TXNDC5 is an ER membrane protein that has protein disulfide isomerase activity. This was significant across all experiments and will need further validating but is unlikely to be biologically meaningful. SCD, an ER protein, is involved in fatty acid biosynthesis but the ratios were not consistent so was ranked as low.

Consistent with its low abundance, we could not quantify PI5P4K2β in the first two replicates. However upon increasing cell numbers, we were able to quantify both isoforms. We therefore used datasets 3 and 4 for mining interactors. We found good correlation of SILAC ratios when comparing the replicates and reciprocals (Fig. 3A, B) and for FLAG pull-downs both PI5P4K2β and PI5P4K2α had statistically significant ratios (> 2 SD from the median) as well as one other protein NT5C2, a cytosolic purine 5-nucleotidase. The TALON data showed alternate proteins that were of greatest significance with the PI5P4K2 being only 1SD from the median ratio. Of the 218 FLAG and 348 TALON purified proteins 84 proteins were identified using the two different affinity tags. We took all proteins with > 1 SD of median from each of the four FLAG and TALON datasets (Supplemental Table 3A&B, Table 3 in [23]) and found that only 18 were in common for both pull-downs. These are listed in Fig. 3C and STRING interaction mapping was performed to analyse the validity of these (Fig. 3D). STRING clearly showed both the PI5P4K2β and PI5P4K2α isoforms as well as NT5C2, TXNDC5 and SCD as non-interaction proteins distinct from a large cluster of mostly metabolic related proteins and a smaller cluster of protein processing proteins (PDIAs and chaperones). To check the validity of these non-interacting proteins we expanded the string network to include increments of ten additional known interactors (Supplemental Fig. S6) and still did not find a connecting protein for PI5P4K2β and PI5P4K2α thus concluding that these isoforms do not interact with any other proteins. For proteins NT5C2, TXNDC5 and SCD we have ranked these as high, medium or low confidence based on the identification and ratio reproducibility amongst replicated, reciprocal and tags (Fig. 3D). NT5C2, a cytosolic protein may have a critical role in the maintenance of a constant composition of intracellular purine/pyrimidine nucleotides in cooperation with other nucleotidases, and preferentially hydrolyses inosine 5′-monophosphate (IMP) and other purine nucleotides. TXNDC5 is an ER membrane protein that has protein disulfide isomerase activity. This was significant across all experiments and will need further validating but is unlikely to be biologically meaningful. SCD, an ER protein, is involved in fatty acid biosynthesis but the ratios were not consistent so was ranked as low.

The enzyme PI5P4K2 acts on lipid substrates, and previous experimental findings indicate no other interactors in this complex [16]. Hence for the case of PI5P4K2, our exhaustive SILAC–iPAC data has successfully identified the one known and biologically relevant binding partner of PI5P4K2β subunit against a large excess of non-specific interactors. Moreover, our data strongly implies that PI5P4K2 does not interact with any other protein. Hence, the enzyme is most likely is localised to the nuclear membrane *via* protein–lipid interactions. When comparing our data with the traditional non quantitative iPAC approach (by searching Mascot with no quantitation) the PI5P4K2 proteins were not ranked highly (120 of 197 hits by Mascot) in identification because of the mass of non-specific proteins but with quantitation we were able to successfully enrich for these and classify most co-purifying proteins as non-specific contaminants. In conclusion there is no evidence that PI5P4K2 proteins interact with other proteins in this lipid environment, consistent with previous reports.

### 3.4 FANCC

Here we carried out two reciprocal labellings, totalling 4 replicates per affinity tag. The bait FANCC and known Fanconi core complex members, FANC-A, B, E, F, G, L, M and FAAP100 were identified in combined replicate datasets for both parallel pulldowns (Fig. 2, supplemental Table S2*B*). The two different affinity purifications resulted in some different peptides being observed, with IgG pulldowns yielding higher percentage sequence coverage of 8.4% for the bait FANCC (Supplemental Fig. S5).

Here we carried out two reciprocal labellings, totalling 4 replicates per affinity tag. The bait FANCC and known Fanconi core complex members, FANC-A, B, E, F, G, L, M and FAAP100 were identified in combined replicate datasets for both parallel pull-downs (Fig. 2, supplemental Table S2*B*). The two different affinity purifications resulted in some different peptides being observed, with IgG pull-downs yielding higher percentage sequence coverage of 8.4% for the bait FANCC (Supplemental Fig. S5).

**Fig. 4:**
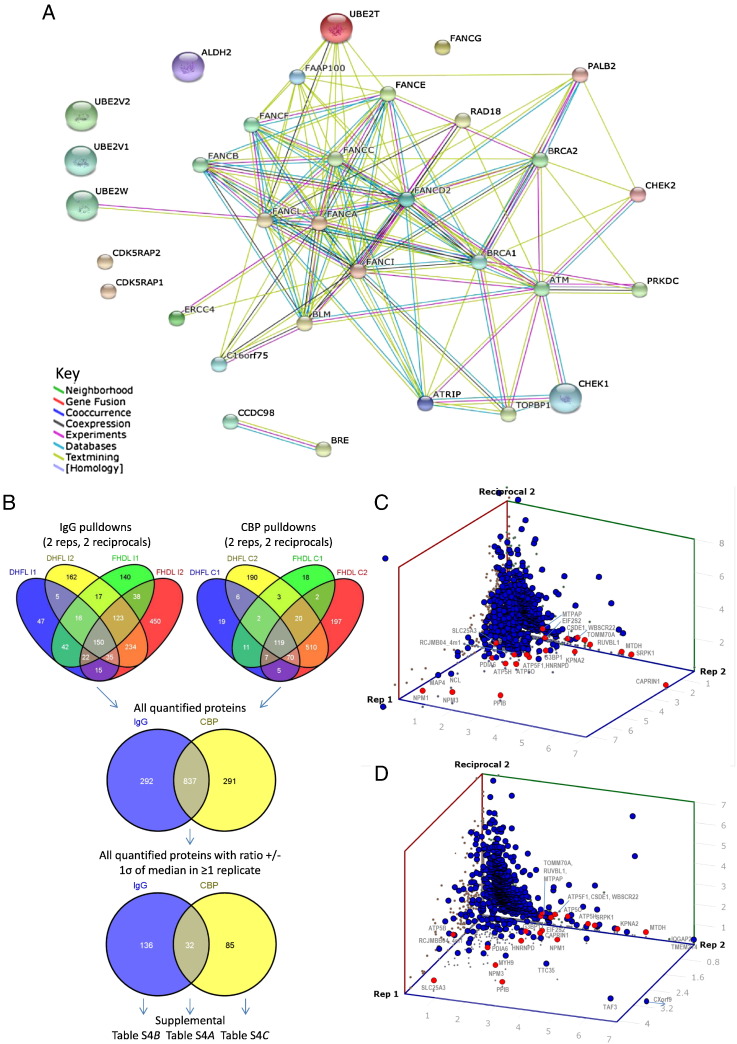

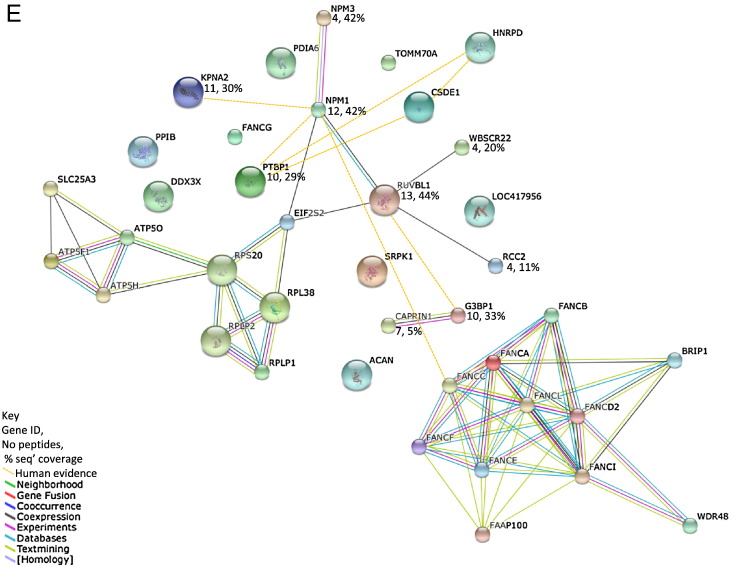
*(A)* STRING interaction map of the known protein candidates present only associated with tagged FANCC using the *Gallus gallus* database. *(B)* Venn diagrams to show replicate reproducibility of all quantified identifications from the two parallel affinity pull-down approaches. *(C)* Scatter plots to show the distribution of protein ratios for the FANCC calmodulin pull-downs for DT40 Heavy SILAC labelling, FANC Light labelling (DHFL) and the reciprocal labelling experiments (FHDL) respectively and *(D)* IgG pull-downs that showed ratios +/− > 1SD from the median ratio. *(E)* STRING interaction map of the newly identified protein candidates in both calmodulin IgG pull-downs. (See Supplemental Fig. S9 for tag specific interactors).

In these experiments, we looked at the FANC complex present in rapidly and exponentially growing cells in which DNA damage had not been artificially initiated. We searched our lists for proteins known to be involved in DNA repair [24,25] and found good representation of known proteins including FANCD2, FANCI, FANCJ, (with 7%, 6.5% and 5.8% sequence coverage respectively). A comprehensive list of known FANC interactors is presented in supplemental Table S3C as well as a STRING interaction map to indicate all experimental and predicted FANC interactions in *G. gallus* (Fig. 4A). Not all were quantified due to low peptide hits in the individual replicates, and those that were, CHEK1; CDK5; UBE2V2, were not statistically significantly enriched for.

In these experiments, we looked at the FANC complex present in rapidly and exponentially growing cells in which DNA damage had not been artificially initiated. We searched our lists for proteins known to be involved in DNA repair [24,25] and found good representation of known proteins including FANCD2, FANCI, FANCJ, (with 7%, 6.5% and 5.8% sequence coverage respectively). A comprehensive list of known FANC interactors is presented in supplemental Table S3C as well as a STRING interaction map to indicate all experimental and predicted FANC interactions in *G. gallus* (Fig. 4A). Not all were quantified due to low peptide hits in the individual replicates, and those that were, CHEK1; CDK5; UBE2V2, were not statistically significantly enriched for.

To search for new interactors we analysed the 837 of 1126 quantified proteins that were common to calmodulin and IgG resins (Fig. 4B) and present the three largest datasets for each affinity resin (Fig. 4C&*D*). As expected the majority of proteins cluster around the 1:1 ratio, corresponding to non-specific interactors, but there were also some notable proteins common to both tags which appeared as highly significant that lay > 1SD from the median ratio (Supplemental Table S4*A*, Table 4 in [23]). Of particular interest were eight nuclear proteins involved in DNA damage, including RUVBL1 (44% sequence coverage by MS). We used STRING to assess the validity of these proteins and showed co-expression evidence of RUVBL1 with WBSC22, RCC, NPM1 and NPM3 (Fig. 4E). The peptide count and % sequence coverage of these proteins was significant (> 2 unique and > 10% respectively) confirming their existence. These are indeed all nuclear localised proteins and may be newly identified interactors, not previously identified in *Gallus*. We also searched these using the equivalent human identifiers and found experimental evidence of NPM1 binding both FANCA and FANCC (Supplemental Fig. 9A). RUVBL1 has been shown to interact with RUVBL2 in other species so we searched our MS data against a bovine database and found up to 19% protein coverage in all replicates with evidence of both heavy and light peptide pairs that would have resulted in quantitation had RUVBL2 not been absent from the Gallus database. With our new quantitative evidence of NPM1 and RUVBL1 interacting with FANCC we have identified a new family of interactors involved in DNA repair in *Gallus* (Fig. 4E).

To search for new interactors we analysed the 837 of 1126 quantified proteins that were common to calmodulin and IgG resins (Fig. 4B) and present the three largest datasets for each affinity resin (Fig. 4C&*D*). As expected the majority of proteins cluster around the 1:1 ratio, corresponding to non-specific interactors, but there were also some notable proteins common to both tags which appeared as highly significant that lay > 1SD from the median ratio (Supplemental Table S4*A*, Table 4 in [23]). Of particular interest were eight nuclear proteins involved in DNA damage, including RUVBL1 (44% sequence coverage by MS). We used STRING to assess the validity of these proteins and showed co-expression evidence of RUVBL1 with WBSC22, RCC, NPM1 and NPM3 (Fig. 4E). The peptide count and % sequence coverage of these proteins was significant (> 2 unique and > 10% respectively) confirming their existence. These are indeed all nuclear localised proteins and may be newly identified interactors, not previously identified in *Gallus*. We also searched these using the equivalent human identifiers and found experimental evidence of NPM1 binding both FANCA and FANCC (Supplemental Fig. 9A). RUVBL1 has been shown to interact with RUVBL2 in other species so we searched our MS data against a bovine database and found up to 19% protein coverage in all replicates with evidence of both heavy and light peptide pairs that would have resulted in quantitation had RUVBL2 not been absent from the *Gallus* database. With our new quantitative evidence of NPM1 and RUVBL1 interacting with FANCC we have identified a new family of interactors involved in DNA repair in *Gallus* (Fig. 4E).

In addition to comparing the reproducibility of all identifications across the four replicates for both Calmodulin and IgG pull-downs (Fig. 4D) we also looked at statistically significant proteins that were observed with only one or other resin (supplemental Tables S4*B*&*C*). Although some nuclear proteins were present, most were cytosolic, endomembrane or mitochondrial, and thus unlikely to be biologically relevant. STRING mapping showed experimental evidence of only a few proteins directly interacting, via FANC-A: brg1/SMARCA4a, a SWI/SNF-related matrix-associated actin-dependent regulator of chromatin proteins for Calmodulin resin (Supplemental Fig. S9*B*) and RPA1 (Ras related) that in turn binds SSRP1, a recombination signal sequence recognition protein 1for IgG resin (Supplemental Fig. S9*C*). These were reproducible and statistically significant and show that not all resins can capture genuine interactors demonstrating that multiple AP-MS approaches are required to capture all complex members.

In addition to comparing the reproducibility of all identifications across the four replicates for both Calmodulin and IgG pull-downs (Fig. 4D) we also looked at statistically significant proteins that were observed with only one or other resin (supplemental Tables S4*B*&*C*). Although some nuclear proteins were present, most were cytosolic, endomembrane or mitochondrial, and thus unlikely to be biologically relevant. STRING mapping showed experimental evidence of only a few proteins directly interacting, via FANC-A: brg1/SMARCA4a, a SWI/SNF-related matrix-associated actin-dependent regulator of chromatin proteins for Calmodulin resin (Supplemental Fig. S9*B*) and RPA1 (Ras related) that in turn binds SSRP1, a recombination signal sequence recognition protein 1 for IgG resin (Supplemental Fig. S9*C*). These were reproducible and statistically significant and show that not all resins can capture genuine interactors demonstrating that multiple AP-MS approaches are required to capture all complex members.

Comparing these significant lists with those obtained using traditional iPAC (by processing data by Mascot with no quantitation) only NPM1 of the newly identified proteins was found and it was also present in the negative control so would have been excluded by iPAC due to the similar numbers of peptides demonstrating that quantitation is necessary.

## 4 Discussion

A major technical challenge in proteomics is how to detect low-abundance binding interactions amid a large excess of non-specific proteins. The SILAC–iPAC method is designed to address this question. Firstly, we use multiple affinity capture carried out in parallel. This approach offers the advantages of reduced incubation times and potentially higher recoveries compared to serial methods such as TAP tagging. Secondly, as technical variability can often be high with AP-MS experiments [26] we use the quantitation provided by SILAC labelling to provide confident discrimination between specific and non-specific interactors. In an ideal and totally clean pull-down, a highly specific interaction between tag and resin will yield the tagged protein that is labelled with either only light or heavy isotopes from non-labelled or SILAC labelled cells respectively. It was in these lists where we found some of our more interesting and potentially biologically relevant proteins for the Fanconi complex. However, high abundance proteins may appear in both samples, and here quantitation will be essential to discriminate the genuine from non-specific binders.

During the course of the work, we became aware of an additional, and under-appreciated source of error in traditional SILAC AP-MS methods [27]. Not all affinity resins bind to the same sets of non-specific proteins. This can make it hard for genuine low abundant binders to compete for an immobilised ligand, thus making quantitation difficult. The severity of the problem varies from resin to resin. Our approach has been to compare proteins from multiple parallel affinity purifications so that we can be sure that we are investigating genuine interactors. In our experiments, we detected some protein with a statistically significant ratio from one resin but not from an alternate resin. We even found statistically significant proteins that were only associated with tagged bait from one affinity resin, but present in both specific and control pull-downs with the second resin with non-significant ratios (depicted in Fig. 1). Such proteins would seem genuine if only a single tag AP experiment is performed. Our data confirm that they are really non-specific interactors and of significant concern. They represent a class of non-specific artefacts that have not been previously emphasised in traditional pull-down experiments.

We deliberately chose two contrasting examples whose interactomes are currently known or uncertain: PIP5P4K2β and FANCC. In the case of PI5P4K2β, other than its homodimeric association in the nucleus, the only other established partner is PI5P4K2α. Under these conditions, PI5P4K2β and its known binding partner PI5P4K2α were successfully identified. The PI5P4K2β pull-downs were highly specific, so that initial experiments failed to detect any PI5P4K2β peptides in the control sample. Only when using two fold higher cell preparations were some non-tagged PI5P4K2β peptides detectable in the mixed SILAC samples. The ratio of PI5P4K2β:PI5P4K2α, calculated at 1.28 was consistent with 1.36 using an internal labelled standard approach [16]. Importantly we were able to detect these interactions using only 1 × 10^8^ cells where previous attempts had to use 2 × 10^10^ cells [16]. No other proteins in the pull-downs were reproducibly detected, and none had ratios that were statistically different from 1:1. We have confirmed this interaction using two independent tags and quantitatively proven that the remaining candidate proteins identified by MS across 16 replicates are, in fact, non-specific contaminants and/or non-reproducible or perhaps transient interactors. Thus there still remains no experimental evidence that PI5P4K2β stably binds to any protein other than PI5P4K2α in DT40 cells. The interaction of PI5P4K2 with the nuclear membrane is therefore likely to occur *via* protein–lipid interactions, rather than protein–protein interactions.

The Fanconi complex was more challenging to address. The complex is prominently associated with stalled replication forks, so isolating enough bait with intact native complexes can be limiting. The FA core is composed of a large multi-protein nuclear complex comprised of the proteins mentioned [18]. Within the core complex are sub-complexes, A&G, C, E & F and B, L and FAAP100 proteins, and together with M, FAAP proteins 10, 16, 20, and 24 this core complex ubiquitinates D2 and FANCI. From multiple replicates we were able to identify not only the Fanconi core groups A, B, C, E, F, G, J, L and M but also their substrates D2 and I, and downstream players FANCD1, N, O, P & Q. A key objective has been to identify new interacting partners for this complex. These interactors may in themselves be new FA proteins and may provide further mechanistic insight into how the pathway works. We have identified proteins involved in the DNA repair pathway, namely kinases ATM, ATR, PRKDC, CHK1&2; BRCA1&2 and known interactors FAM175, BRE, PALB2 (FANCN), and BLM; endonucleases ERCC4, EME1 and SLX4 (FANCP); CDK5 and its regulatory proteins CDKRAP1&2; ubiquitin conjugating enzyme E2 family Ube2T; and FAN1. Many of these are transient and/or low abundance proteins, and present at different stages of the pathway, and were all enriched for in our pull-downs.

Addressing abundant proteins using SILAC again allowed us to distinguish the genuine binders from the mass of non-specific proteins. Interesting DNA-binding proteins that were common in both affinity methods were RUVBL1, a TATA box-binding protein-interacting protein, WBSCR22 involved in DNA methylation, RCC2, a telophase disk protein and NMP1 (Nucleophosmin), a nucleolar phosphoprotein B23 involved in genomic stability and DNA repair amongst other roles. STRING showed evidence of RUVBL1 interacting with all these proteins (Fig. 4). Importantly RUVBL1 has recently been independently characterised to interact with the FA complex, serving a role in DNA crosslink repair using functional readouts [28]. It should be emphasised that our method detected RUVBL1 with up to 10^3^ fold lower cell preparations. Therefore, SILAC–iPAC offers significant practical advantages in terms of sensitivity and quantitative analysis. We also found equivalent proportions of the RUVBL1 interacting partner RUVBL2 when searching our data using a bovine database. This was also functionally confirmed by Rajendra et al. [28]. We also identified ATPase subunits which according to STRING are indirect interactors of RUVBL1-FA through a cluster of ribosomal proteins, all of which were abundant proteins in our DT40 parts lists. When searching our list against human interactions we found evidence of both FANCA and C binding NPM1 that co-purifies with EIF2S2, which interacts with many ribosomal proteins that also associate with ATP synthesis machinery. Other enriched proteins such as CAPRIN1, a cell cycle associated protein, and G3BP1, a DNA-unwinding enzyme, are of potential biological interest and will have to be independently confirmed.

**Fig. 5:**
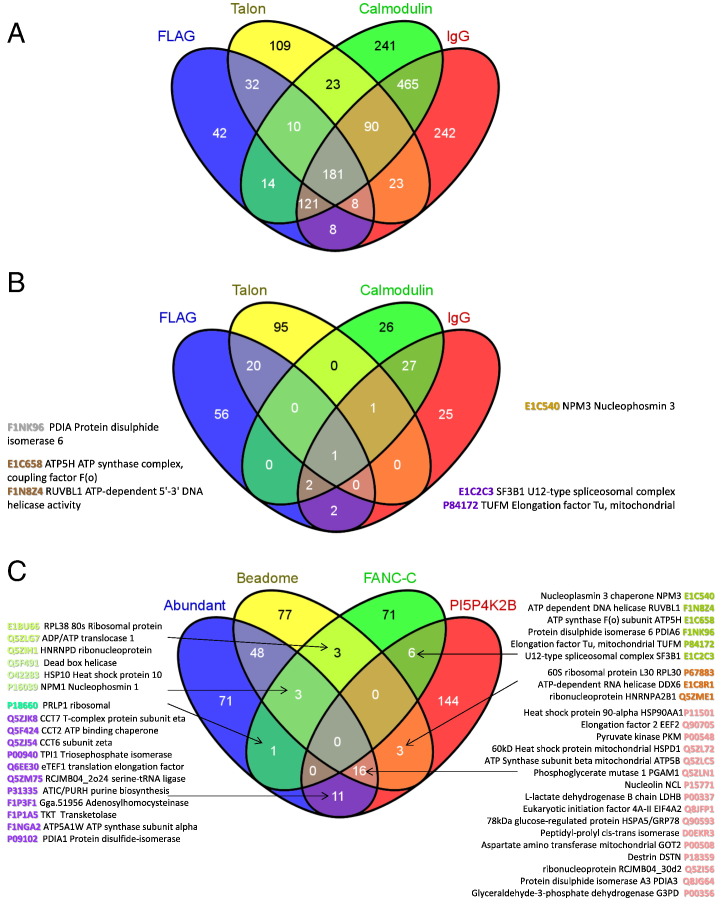
Venn diagrams to show the comparison of interaction proteins from two different complexes, PI5P4K2 and FANC, *(A)* total hits, *(B)* statistically significant, and *(C)* a comparison with the DT40 abundant proteins and previously identified non-specific binders to ≥ 2 of the four resins used (beadome).

With two different complex models being studied here we were interested in seeing if any identified and significant proteins appeared in each other's pull-down lists (Fig. 5A and *B* respectively). Using data from four replicates each for FLAG, TALON, Calmodulin and IgG pull-downs, of all identified and quantified proteins 181 (11.2%) were present in all four different affinity pull-downs with a further 229 (14.2%) present in three resins and 68 (4.2%) from two resins that were not from the same bait. In comparison 30.9% of all proteins were from parallel datasets with the same tag (Fig. 5A). Taking only the proteins that had at least one significant ratio in any one replicate, the proportion of significant proteins in both the parallel pull-downs was vastly increased by comparing only statistically significant ratios and the overlap across all resins was greatly reduced (Fig. 5B) and comprised only PDIA in all four resins and ATP5H, NPM3 and RUVBL1 in three of four, and in these three cases the proportion of significant ratios was higher in the FANCC pull-downs where we found published experimental evidence of interactions. It is possible that chaperones such as PDIA are recruited to endogenously tagged proteins to assist in folding. This highlights the fact that even stringent filtering measures can result in promiscuous proteins supposedly interacting and hence the need for published beadomes [2,10]. Without suitable antibodies for such a large number of candidates it would be unfeasible to prove if they are indeed genuine.

Lastly we compared the statistically significant proteins from both complex models to the abundant DT40 protein list and the lists of sticky proteins observed with two or more different resins (beadome) (Fig. 5C) and reassuringly no proteins were common to all sets. Of the significant FANCC candidates 85% were unique to FANCC pull-downs and not identified in any abundant or beadome lists. The significant RUVBL1 was absent from abundant or beadome lists but did co-purify with PI5P4K2β from FLAG resins. NPM1 pulled down with FANCC was present in the abundant and beadome lists but they were reassuringly absent from the PI5P4K2β lists. Most other proteins in common to both complexes and the beadome were protein processing proteins, such as chaperones, that could be genuine transient interactors whilst the bait proteins are synthesised and trafficked.

Our initial survey to ascertain abundant DT40 proteins from a crude lysate to establish likely non-specific contaminants showed that almost half were reproducibly identified in the four DT40 ‘beadomes’. These individual beadomes will be useful resources for the community in planning targeted PPI studies and have been deposited in the forthcoming release of the CRAPome repository. These comprise the first reports of chicken data and findings are consistent with other beadome lists from human yeast and *Drosophila*.

Although the chicken DT40 cell line has been extensively used in performing homologous recombination to endogenously tag proteins of choice, one major disadvantage is that the chicken genome and proteome databases are poorly maintained and annotated, and there is an unacceptable level of redundancy. This makes the identification of unique peptides unnecessarily problematic. We had to use the UniprotKB database with TrEMBL entries as some of our bait and known interactors did not have SwissProt entries and this significantly increases proteome redundancy. For example, the UniprotKB 2012 release contained 31,529 protein entries and the 2013 release had a 7% increase to 33,804 entries for only 16,294 genes. Approximately 20,000 (~ 60%) of these proteins were computationally predicted. Similarly the ‘reference proteome’ with 23,395 *Gallus* only entries comprise only 9.6% SwissProt entries and the remainder are TrEMBL of which most are predicted. In addition a significant proportion of protein entries in the UniprotKB database are represented by multiple accession numbers with one example having 20 that included fragments of the same protein. Whilst various quantitation software packages have the option of grouping similar proteins according to homology it was difficult to quantify, for example, the distinct PI5P4K2β and α isoforms due to their high sequence similarity, thereby making reliable quantitation challenging. In addition when investigating genuine binding partners that did not appear in controls, protein scoring can be misleading with a redundant database. In addition to redundancy there is evidence of incompleteness such as the RUVBL2 protein that we identified with 25% coverage when searching other organisms' databases. With an increasing number of researchers using the DT40 cell line and chicken as a model organism for immunology and embryology and in analysis of human disease [9,29], efforts must be made to consolidate and update these databases.

Compared to other methods SILAC is generally the method of choice for quantitative AP-MS experiments [3] and allows proteins with ratios deviating from 1:1 to be pursued. Whilst many other quantitative interactome studies using AP combined with other isobaric tags such as iTRAQ and TMT can discriminate between specific interacting partners and non-specific binding contaminants, the addition of parallel APs combined with SILAC provides another level of assurance to our quantitative method. Our results indicate that SILAC–iPAC will be useful for determining genuine complex binding partners as well as cataloguing non-specific proteins that bind to specific or multiple affinity resins. It can be used for cultured cells, or organisms that can be metabolically labelled that have proteins tagged either endogenously or exogenously. This will make the method widely applicable to the proteomics community.

The following are the supplementary data related to this article.

Supplemental Fig. S1. Mass spectrometry data workflow and protein acceptance criteria.
Supplemental Fig. S2. GO annotation and proteins over- and under-represented in DT40 lysates to determine abundance and potential contaminants. *(A*) Pie chart showing the GO cellular components classification of DT40. *(B*) Proteins under-represented in DT40 compared to the *G. gallus* SwissProt proteome. *(C)* Proteins over-represented in DT40 compared to the *G. gallus* SwissProt proteome.
Supplemental Fig. S3. Non-specific binding proteins associated with ≥ 2 of 4 affinity resins: FLAG, TALON, Calmodulin and IgG sepharose. *(A)* Pie chart showing GO_Cellular component annotation. *(B)* GO_Molecular function annotation and *(C)* Kegg pathway analysis. ‘Other’ represents proteins other categories with single protein entries. Data analysed using ProteinCenter. *(D)* Overlap of proteins identified as the abundome and beadome.
Supplemental Fig. S4. PI5P4K2α and β alignment. PI5P4K2α has unique swissprot (spQ5F356) and trembl (trF1NKL5) accessions, both included in UniprotKB *G. gallus* database 2012. Highlighted in purple and blue are unique peptides to PI5P4K2 α and β respectively, and in yellow are conserved peptides identified in replicate SILAC–iPAC screenings. Green are positively charged residues where K and L indicate ends of peptides that can be quantified.
Supplemental Fig. S5. A*.*Fanconi anaemia complementation group C *G. gallus* UniprotKB accession alignment with peptides identified in both Calmodulin and IgG pull-downs replicate SILAC–iPAC screenings highlighted in green and IgG only in orange.
Supplemental Fig. S6. Expanded PIP4Kin2 STRING network (+ 10) to identify potential missing interactors in our lists.
Supplemental Fig. S7. All TALON interactors based on experimental evidence only.
Supplemental Fig. S8. All FLAG interactors based on experimental evidence only.
Supplemental Fig. S9. Proteins interacting with FA proteins using human accessions (*A*), Gallus accessions specific to only Calmodulin (*B*) or IgG (*C*) resins.

Supplemental Table S1 *(A)* The top 150 abundant proteins from 3 replicate DT40 lysate analyses, ranked by emPAI score. *(B)* The top 150 DT40 proteins identified that bind non-specifically to the resins, FLAG, TALON, Calmodulin and IgG, used in this study.

Supplemental Table S2 Peptides identified using Mascot Percolator for *(A)* PI5P4K2β pull-downs and *(B)* FANCC pull-downs. *(C)* Known DNA repair proteins identified in FANCC pull-downs.

Supplemental Table S3 PI5P4K2β associated statistically significant proteins from replicate datasets that were pulled down with FLAG resins *(A)* or TALON *(B)* resins with proteins in common highlighted in green.

Supplemental Table S4 FANCC associated proteins that had at least one statistically significant protein from replicate datasets that were pulled down with both calmodulin and IgG pull-downs *(A)*, calmodulin resins *(B)* or IgG resins only *(C)*.

Supplementary data to this article can be found online at http://dx.doi.org/10.1016/j.jprot.2014.12.006.

## Conflict of interest

None
