# Supplementary material for: SILAC-iPAC: A quantitative method for distinguishing genuine from non-specific components of protein complexes by parallel affinity capture
Source: J Proteomics. 2015 Feb 6;115:143–56. doi: 10.1016/j.jprot.2014.12.006 (PMC4329988; doi:10.1016/j.jprot.2014.12.006)

Supplemental Fig. S1. Mass Spectrometry data workflow and protein acceptance criteria.

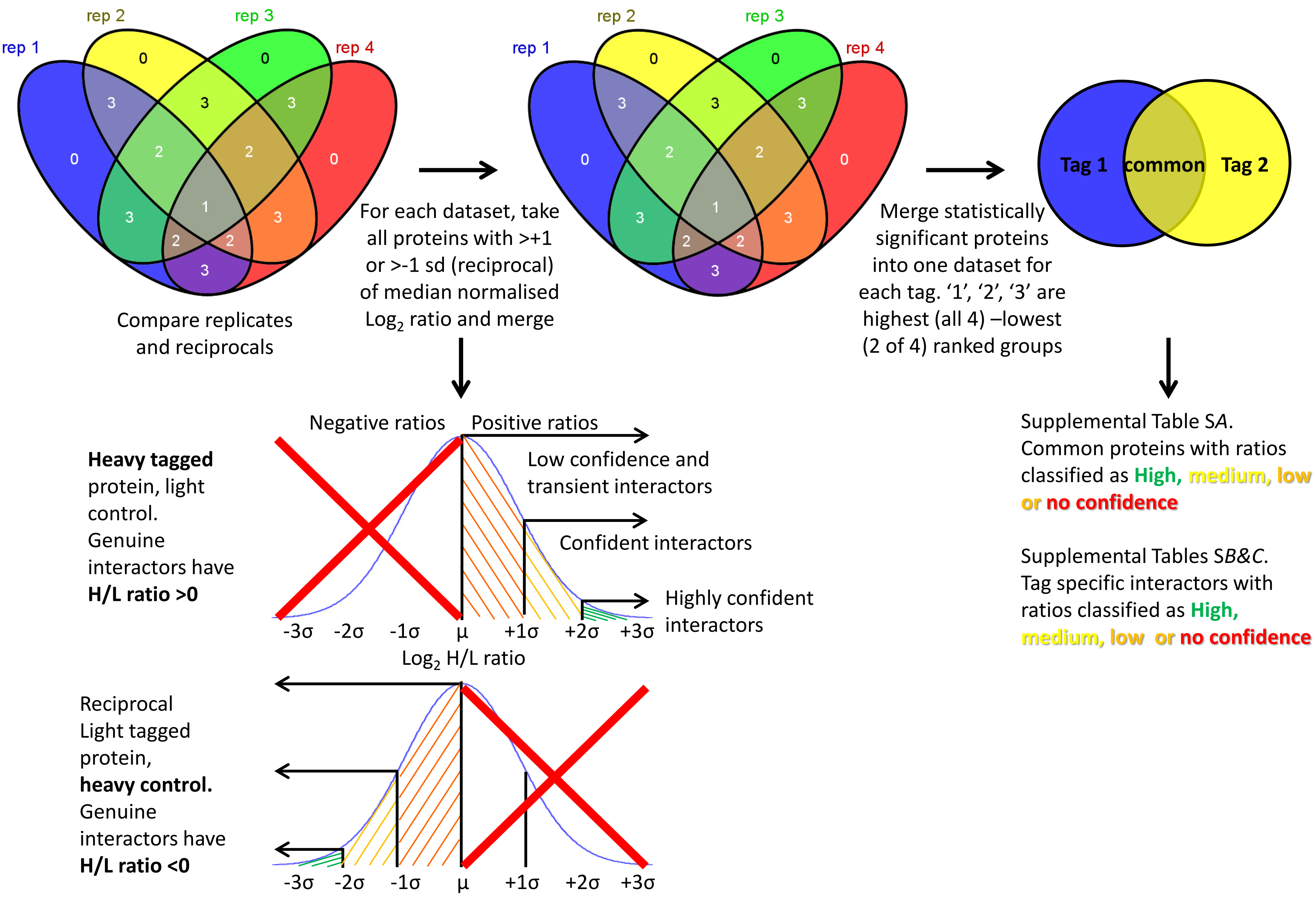

Supplemental Fig. S2. GO annotation and proteins over- and under-represented in DT40 lysate.

A) Pie chart showing the GO cellular components classification of DT40

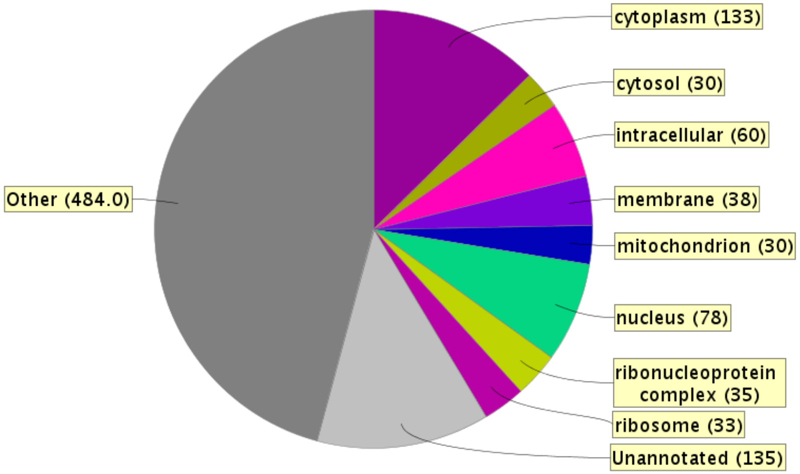

B) Proteins under-represented in DT40 compared to the *Gallus gallus* SwissProt proteome

Description	Count [%]	Ref. Count [%]	Count	Ref. Count	Raw p-value	FDR p-value
cellular_component	73.38	89.85	306	2143	7.29E-28	3.99E-24
intrinsic to membrane	6.95	22.39	29	534	4.20E-20	3.28E-17
integral to membrane	6.95	21.3	29	508	4.17E-18	1.14E-15
extracellular region	2.16	12.87	9	307	5.62E-17	1.28E-14
membrane	17.75	33.88	74	808	9.93E-16	2.05E-13
membrane part	12.47	25.83	52	616	2.70E-13	4.62E-11
plasma membrane	5.04	14.68	21	350	1.18E-11	1.90E-09
cell periphery	6.24	15.51	26	370	2.92E-10	4.31E-08
nucleus	21.1	33	88	787	3.29E-09	4.61E-07
intracellular membrane-bounded organelle	34.53	46.96	144	1120	1.14E-08	1.52E-06
membrane-bounded organelle	34.53	46.96	144	1120	1.14E-08	1.52E-06
extracellular matrix	0	3.35	0	80	1.58E-07	1.96E-05
proteinaceous extracellular matrix	0	3.1	0	74	5.21E-07	5.82E-05
extracellular region part	1.68	6.16	7	147	1.98E-06	2.04E-04
chromosomal part	0.96	4.07	4	97	5.37E-05	3.65E-03
chromosome	1.44	4.86	6	116	5.40E-05	3.65E-03
cell junction	0.72	3.4	3	81	1.41E-04	8.45E-03
cell	66.19	73.5	276	1753	1.60E-04	9.14E-03
cell part	66.19	73.5	276	1753	1.60E-04	9.14E-03
extracellular matrix part	0	1.59	0	38	6.32E-04	2.91E-02
chromatin	0.24	2.1	1	50	7.13E-04	3.20E-02
synapse	0.24	2.1	1	50	7.13E-04	3.20E-02
plasma membrane part	2.16	5.12	9	122	8.39E-04	3.70E-02

C) Proteins over-represented in DT40 compared to the *Gallus gallus* SwissProt proteome

Description	Count [%]	Ref. Coun	Count	Ref. Coun	Raw p-value	FDR p-valu
ribosome	7.91	1.34	33	32	<4.90E-324	<4.90E-324
virion	3.12	0.13	13	3	<4.90E-324	<4.90E-324
virion part	3.12	0.13	13	3	<4.90E-324	<4.90E-324
viral capsid	2.88	0.04	12	1	<4.90E-324	<4.90E-324
cytosolic large ribosomal subunit	1.68	0.25	7	6	<4.90E-324	<4.90E-324
proteasome core complex	1.68	0.13	7	3	<4.90E-324	<4.90E-324
clathrin coat	1.2	0.13	5	3	<4.90E-324	<4.90E-324
proteasome core complex, alpha-subunit complex	0.96	0.08	4	2	<4.90E-324	<4.90E-324
proton-transporting ATP synthase complex, catalytic core F(1)	0.96	0.04	4	1	<4.90E-324	<4.90E-324
chaperonin-containing T-complex	0.72	0	3	0	<4.90E-324	<4.90E-324
clathrin coat of coated pit	0.48	0.04	2	1	<4.90E-324	<4.90E-324
clathrin coat of trans-Golgi network vesicle	0.48	0	2	0	<4.90E-324	<4.90E-324
clathrin vesicle coat	0.48	0	2	0	<4.90E-324	<4.90E-324
septin complex	0.48	0.04	2	1	<4.90E-324	<4.90E-324
trans-Golgi network transport vesicle	0.48	0	2	0	<4.90E-324	<4.90E-324
trans-Golgi network transport vesicle membrane	0.48	0	2	0	<4.90E-324	<4.90E-324
fatty acid beta-oxidation multienzyme complex	0.24	0	1	0	<4.90E-324	<4.90E-324
mitochondrial fatty acid beta-oxidation multienzyme complex	0.24	0	1	0	<4.90E-324	<4.90E-324
mitochondrial proton-transporting ATP synthase complex, catalytic core F(1)	0.24	0	1	0	<4.90E-324	<4.90E-324
perikaryon	0.24	0	1	0	<4.90E-324	<4.90E-324
ribonucleoside-diphosphate reductase complex	0.24	0	1	0	<4.90E-324	<4.90E-324
ribosomal subunit	0.24	0	1	0	<4.90E-324	<4.90E-324
signal recognition particle receptor complex	0.24	0	1	0	<4.90E-324	<4.90E-324
ribonucleoprotein complex	10.07	2.98	42	71	1.27E-15	8.47E-15
ribosomal subunit	3.84	0.67	16	16	5.99E-13	3.87E-12
cytosolic part	4.32	1.05	18	25	2.44E-09	1.52E-08
cytosol	10.55	4.82	44	115	4.54E-08	2.76E-07
large ribosomal subunit	2.16	0.38	9	9	1.42E-07	8.34E-07
proteasome complex	2.16	0.38	9	9	1.42E-07	8.34E-07
cytosolic ribosome	2.64	0.55	11	13	2.32E-07	1.34E-06
macromolecular complex	29.5	20.46	123	488	7.79E-07	4.40E-06
cytoplasmic part	35.01	25.49	146	608	1.14E-06	6.41E-06
cytoplasm	52.28	42.22	218	1007	3.41E-06	1.89E-05
small ribosomal subunit	1.68	0.29	7	7	4.79E-06	2.61E-05
coated membrane	1.68	0.34	7	8	3.25E-05	1.71E-04
membrane coat	1.68	0.34	7	8	3.25E-05	1.71E-04
Golgi-associated vesicle	1.2	0.21	5	5	1.60E-04	8.00E-04
Golgi-associated vesicle membrane	1.2	0.21	5	5	1.60E-04	8.00E-04
proton-transporting ATP synthase complex	0.96	0.17	4	4	9.23E-04	3.99E-03
vesicle coat	0.96	0.17	4	4	9.23E-04	3.99E-03
intracellular non-membrane-bounded organelle	21.58	16.44	90	392	1.47E-03	6.21E-03
non-membrane-bounded organelle	21.58	16.44	90	392	1.47E-03	6.21E-03
proton-transporting two-sector ATPase complex	1.68	0.5	7	12	1.68E-03	7.07E-03
coated vesicle membrane	1.44	0.42	6	10	3.08E-03	1.28E-02
coated vesicle	1.68	0.55	7	13	3.10E-03	1.29E-02
proton-transporting two-sector ATPase complex, catalytic domain	0.96	0.21	4	5	3.98E-03	1.61E-02
AP-type membrane coat adaptor complex	0.72	0.13	3	3	5.31E-03	2.06E-02
clathrin adaptor complex	0.72	0.13	3	3	5.31E-03	2.06E-02
COPI coated vesicle membrane	0.72	0.13	3	3	5.31E-03	2.06E-02
COPI-coated vesicle	0.72	0.13	3	3	5.31E-03	2.06E-02
phosphopyruvate hydratase complex	0.72	0.13	3	3	5.31E-03	2.06E-02
cytoskeletal part	9.35	6.37	39	152	5.70E-03	2.18E-02

Supplemental Fig. S3. Non-specific binding proteins associated with ≥2 of 4 affinity resins: FLAG, Talon, Calmodulin and IgG sepharose. *A*) Pie chart showing GO_Cellular component annotation. *B*) GO_Molecular function annotation and *C*) Kegg pathway analysis. 'Other' represents proteins other categories with single protein entries. Data analysed using ProteinCenter. *D*) Overlap of proteins identified as top 150 abundant and the top 150 beadome.

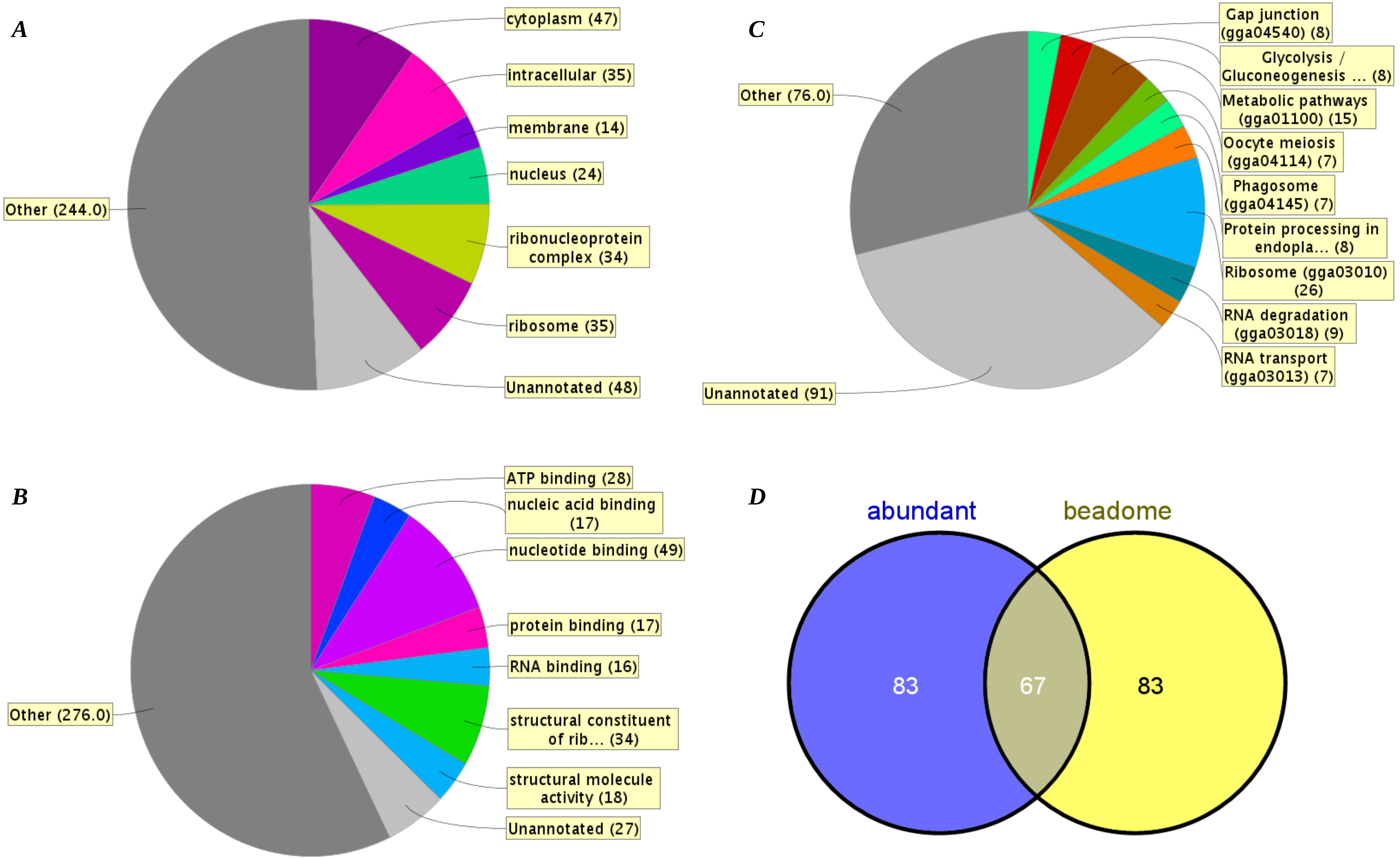

Supplemental Fig. S4. PI5P4K2α and β alignment. PI5P4K2α has unique swissprot (spQ5F356) and trembl (trF1NKL5) accessions, both included in UniprotKB *Gallus gallus* database 2012. Highlighted in purple and blue are unique peptides to PI5P4K2 α and β respectively, and in yellow are conserved peptides identified in replicate SILAC-iPAC screenings. Green are positively charged residues where K and L indicate ends of peptides that can be quantified.

```
1     ------------------------------------AAWVWVLGHNINELSNVPVPVML   23   F1NLK4  β subunit
1     ----------------------------------------------INELSHVQIPVML   13   F1NKL5  α subunit
1     MAAPGTVASVMASKTKTKKKHFVVQKVKLFRASDPLLSVLMWGVNHSINELSHVQIPVML  60   Q5F356  α subunit
                                                ***** .* :****

24    MPDDFKAYSKIKVDNHLFNKENLPSRFKFKEYCPLVFRNLRERFGIDDQDYQNSVTRSAP  83   F1NLK4
14    MPDDFKAYSKIKVDNHLFNKENMPSHFKFKEYCPMVFRNLRERFGIDDQDFQNSLTRSAP  73   F1NKL5
61    MPDDFKAYSKIKVDNHLFNKENMPSHFKFKEYCPMVFRNLRERFGIDDQDFQNSLTRSAP  120  Q5F356
      **********************:**:*********:***************:***:*****

84    VNSDSQGRCGARFLTTYDRRFVIKAVSSEDVAEMHNILKKYHQFIVECHGNTLLPQFLGM  143  F1NLK4
74    LANDSQARSGARFHTSYDKRYIIKTITSEDVAEMHNILKKYHQFIVECHGNTLLPQFLGM  133  F1NKL5
121   LANDSQARSGARFHTSYDKRYIIKTITSEDVAEMHNILKKYHQFIVECHGNTLLPQFLGM  180  Q5F356
      : .***.*.**** *:**:*::**:::********************************

144   YRLTVDGVETYMVVTRNVFSHRLTVHRKYDLKGSTVSREASDKEKAKDLPTFKDNDFLNE  203  F1NLK4
134   YRLTVDGVEIYMIVTRNVFSHRLSVYRKYDLKGSTVAREASDKEKAKELPTFKDNDFIND  193  F1NKL5
181   YRLTVDGVEIYMIVTRNVFSHRLSVYRKYDLKGSTVAREASDKEKAKELPTFKDNDFIND  240  Q5F356
      ********* **:**********:*:**********:**********:*********:*:

204   GQKLHVGEESKKNFLEKLKRDVEFLAQLKIMDYSLLVGIHDVDRAEQEEMEVEDRAEDEE  263  F1NLK4
194   GQKIHIDENNKRMFLEKLKKDVEFLAQLKLMDYSLLVGIHDVERAEQEEVECEENDGEDE  253  F1NKL5
241   GQKIHIDENNKRMFLEKLKKDVEFLAQLKLMDYSLLVGIHDVERAEQEEVECEENDGEDE  300  Q5F356
      ***:*: *:.*: ******:**********:************:******:* *:.  ::*

264   CENDGLGGNPISSYGTPPDSPGNLLNYPRFFGPGEFDPSVDVYAMKSHDSAPKKEVYFMA  323  F1NLK4
254   GESDG-----THPIGTPPDSPGNTLNSSLPLAPGEFDPAIDVYGIKSHESAPRKEVYFMA  308  F1NKL5
301   GESDG-----THPIGTPPDSPGNTLNSSLPLAPGEFDPAIDVYGIKSHESAPRKEVYFMA  355  Q5F356
       *.**        ********* **     :.******::***.:***:***:*******

324   IIDILTPYDAKKKAAHAAKTVKHGAGAEISTVNPEQYSKRFNEFMSNILT  373  F1NLK4   F1NLK4  β subunit
309   IIDILTHYDAKKKAAHAAKTVKHGAGAEISTVNPEQYSKRFLDFIANILT  358  F1NKL5   F1NKL5  α subunit
356   IIDILTHYDAKKKAAHAAKTVKHGAGAEISTVNPEQYSKRFLDFIANILT  405  Q5F356   PI42A_  α subunit
      ****** **********************************  :*::****
```

Supplemental Fig. S5. *A.* Fanconi anemia complementation group C *Gallus gallus* UniprotKB accessions alignment with peptides identified in both Calmodulin and IgG pulldowns replicate SILAC-iPAC screenings highlighted in green and IgG only in orange.

```
1     MAQDTTVPKLNFKFWLDKAIEWGQDTTLESQKDVCLHLPQLQEFLHQTYETLKHMNSIAA   60   Q5F3H0
1     MAQDTTVPKLNFKFWLDKAIEWGQATTLESQKDVCLHLPQLQEFLHQTYETLKHMNSIAA   60   Q60GM0
1     MAQDTTVPKLNFKFWLDKAIEWGQATTLESQKDVCLHLPQLQEFLHQTYETLKHMNSIAA   60   F1NYH1
      ************************ ***********************************

61    IQQFPLIGQLLGRLCWNPFVVGYDESQKTLMWCLCCLYSSEPQNPVELKANSWIRSLLCH   120  Q5F3H0
61    IQQFPLIGQLLGRLCWNPFVVGYDESQKTLMWCLCCLYSSEPQNPVELKANSWIRSLLCH   120  Q60GM0
61    IQQFPLIGQLLGRLCWNPFVVGYDESQKTLMWCLCCLYSSEPQNPVELKANSWIRSLLCH   120  F1NYH1
      ************************************************************

121   LLSSSKWERIEAETSTFISALGYTSADYYCHLVENIVSSLVTELRENQFNELNIQERISV   180  Q5F3H0
121   LLSSSKWERIEAETSTFISALGYTSADYYCHLVENIVSSLVTELRENQFNELNIQERISV   180  Q60GM0
121   LLSSSKWERIEAETSTFISALGYTSADYYCHLVENIVSSLVTELRENQFNELNIQERISV   180  F1NYH1
      ************************************************************

181   SRVNAVSLFCLPLITLPDLTPLLETLLLYHGGASKEILSSEFLEAVNDAVLKKKISLSES   240  Q5F3H0
181   SRVNAVSLFCLPLITLPDLTPLLETLLLYHGGASKEILSSEFLEAVNDAVLKKKISLSES   240  Q60GM0
181   SRVNAVSLFCLPLITLPDLTPLLETLLLYHGGASKEILSSEFLEAVNDAVLKKKISLSES   240  F1NYH1
      ************************************************************

241   AIFSLWHRHLPSLESATLSLLDQLTSLQINSLEKVACVIKDSLLPQAASHPAIFRIVNEI   300  Q5F3H0
241   AIFSLWHRHLPSLESATLSLLDQLTSLQINSLEKVACVIKDSLLPQAASHPAIFRIVNEI   300  Q60GM0
241   AIFSLWHRHLPSLESATLSLLDQLTSLQINSLEKVACVIKDSLLPQAASHPAIFRIVNEI   300  F1NYH1
      ************************************************************

301   FKNALLETDGAPEVMTIIQVFIQLFIQVQQNENKQHKFPLKAYFPHHHQPLVIALLRRPF   360  Q5F3H0
301   FKNALLETDGAPEVMTIIQVFIQLFIQVQQNENKQHKFPLKAYFPHHHQPLVIALLRRPF   360  Q60GM0
301   FKNALLETDGAPEVMTIIQVFIQLFIQVQQNENKQHKFPLKAYFPHHHQPLVIALLRRPF   360  F1NYH1
      ************************************************************

361   ELPSTHWLQHLKHISDTLKSIVEDTNVSSFADLYEIWFLVACFGEWLDIAAEQLMKAAVA   420  Q5F3H0
361   ELPSTHWLQHLKHISDTLKSIVEDTNVSSFADLYEIWFLVACFGEWLGYCTKQLMKAAVA   420  Q60GM0
361   ELPSTHWLQHLKHISDTLKSIVEDTNVSSFADLYEIWFLVACFGEWLDIAAEQLMKAAVA   420  F1NYH1
      ***********************************************  .::********

421   PDALLWLLAFYYCPQNENQQRTQTMVESQAVYSRLMMLYNRTVLSFGDLQASVNNAADTE   480  Q5F3H0
421   PDALLWLLAFYYCPQNENQQRTQTMVESQAVYSRLMMLYNRTVLSFGDLQASVNNAADTE   480  Q60GM0
421   PDALLWLLAFYYCPQNENQQRTQTMVESQAVYSRLMMLYNRTVLSFGDLQASVNNAADTE   480  F1NYH1
      ************************************************************

481   QCHTQCLITHLLTNFLLFSSGGHMIAQEFISHITETTDMSKEVCSLLIRTAYRIKHNGGE   540  Q5F3H0
481   QCHTQCLITHLLTNFLLFSSGGHMIAQEFISHITETTDMSKEVCSLLIRTAYRIKHNGGE   540  Q60GM0
481   QCHTQCLITHLLTNFLLFSSGGHMIAQEFISHITETTDMSKEVCSLLIRTAYRIKHNGGE   540  F1NYH1
      ************************************************************

541   NQRTVKLLNELLQKLAVEV   559  Q5F3H0     Q5F3H0_CHICK
541   NQRTVKLLNELLQKLAVEV   559  Q60GM0     Q60GM0_CHICK
541   NQRTVKLLNELLQKLAVEV   559  F1NYH1     F1NYH1_CHICK
      *******************
```

Supplemental Fig. S6. Expanded PIP4Kin2 STRING network (+10) to identify potential missing interactors in our lists

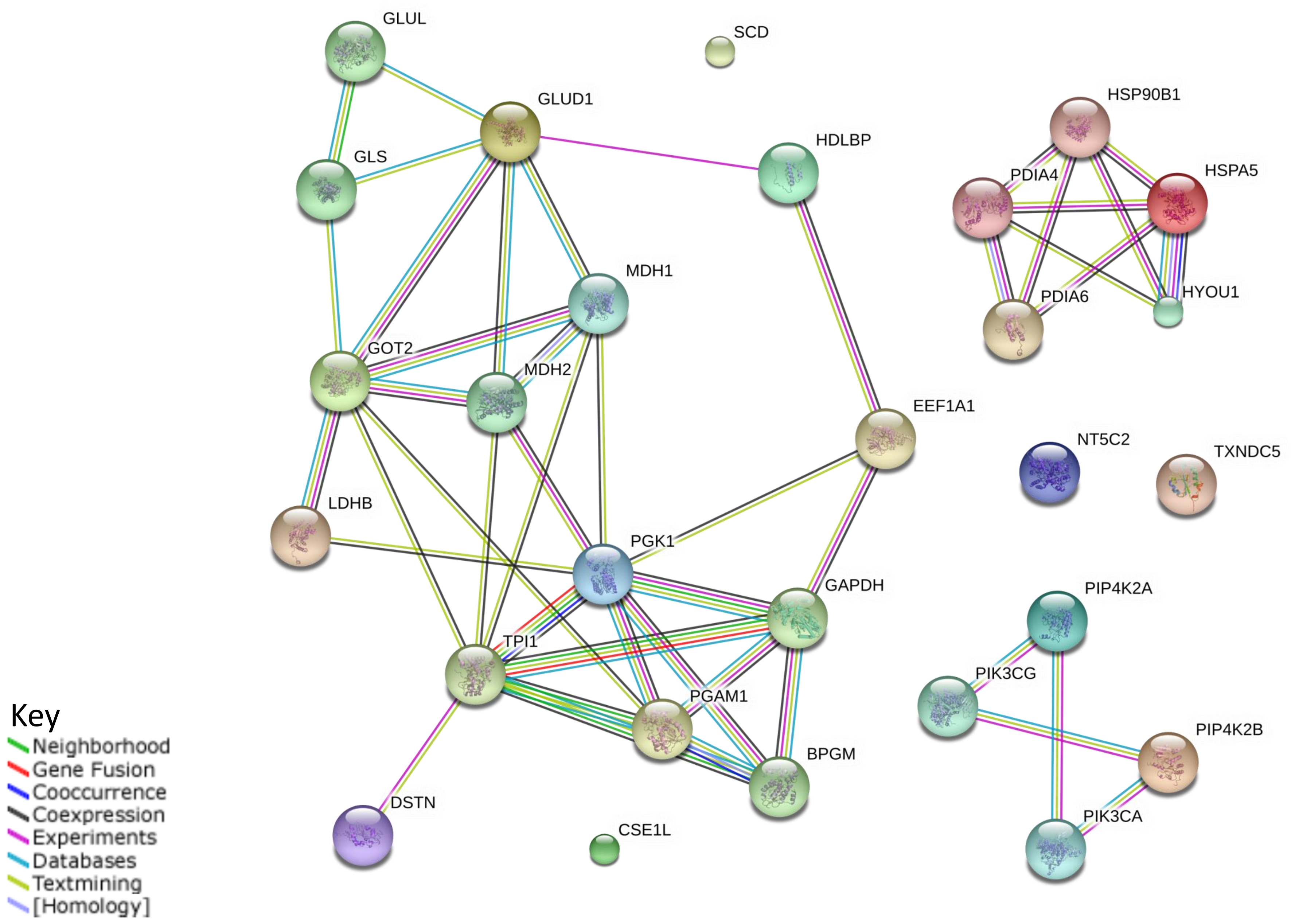

Supplemental Fig. S7. All TALON interactors based on experimental evidence only.

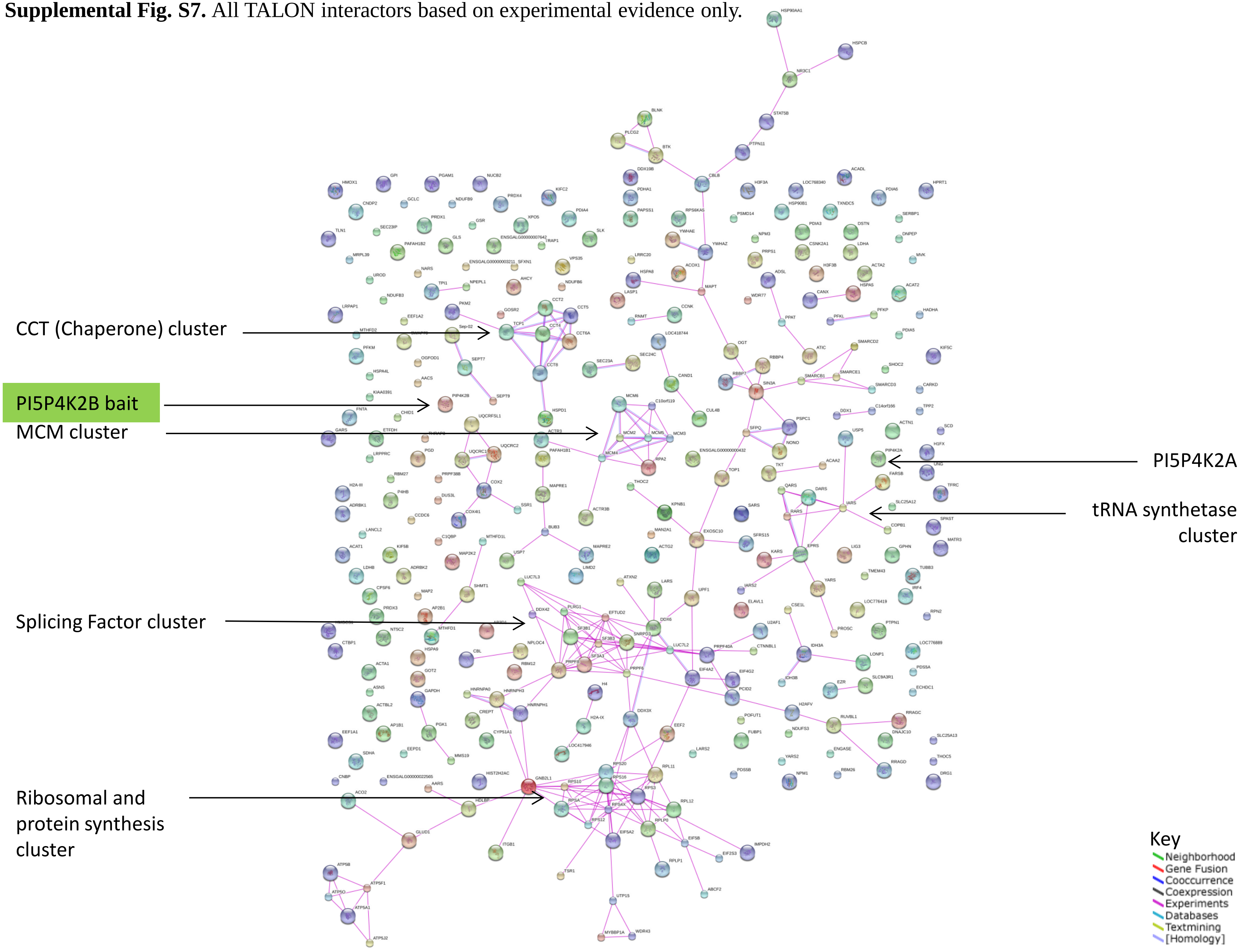

Supplemental Fig. S8. All FLAG interactors based on experimental evidence only.

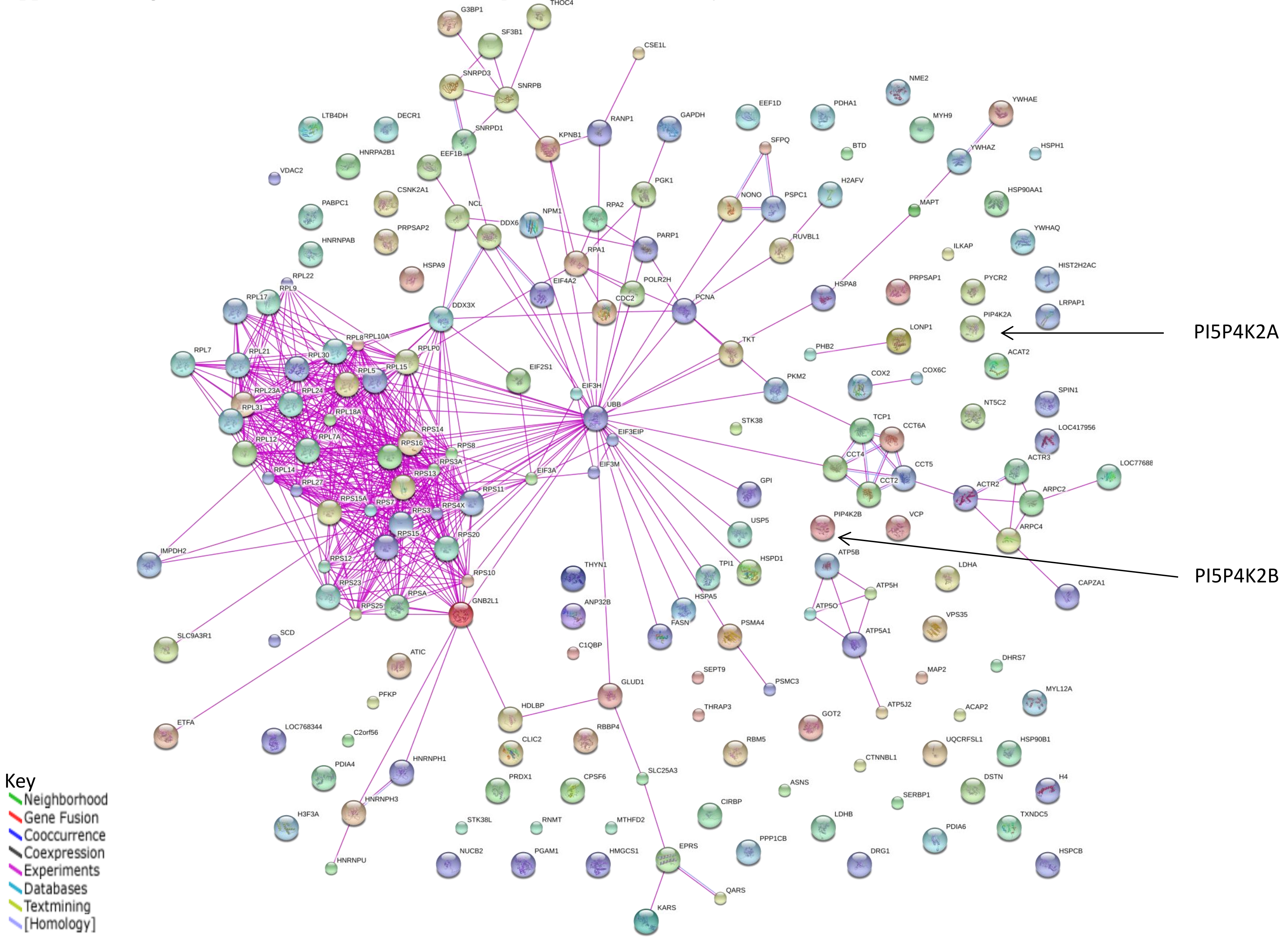

Supplemental Fig. S9. Proteins interacting with FA proteins using human accessions *(A)*, *Gallus* accessions specific to only Calmodulin (*B*) or IgG (*C*) resins

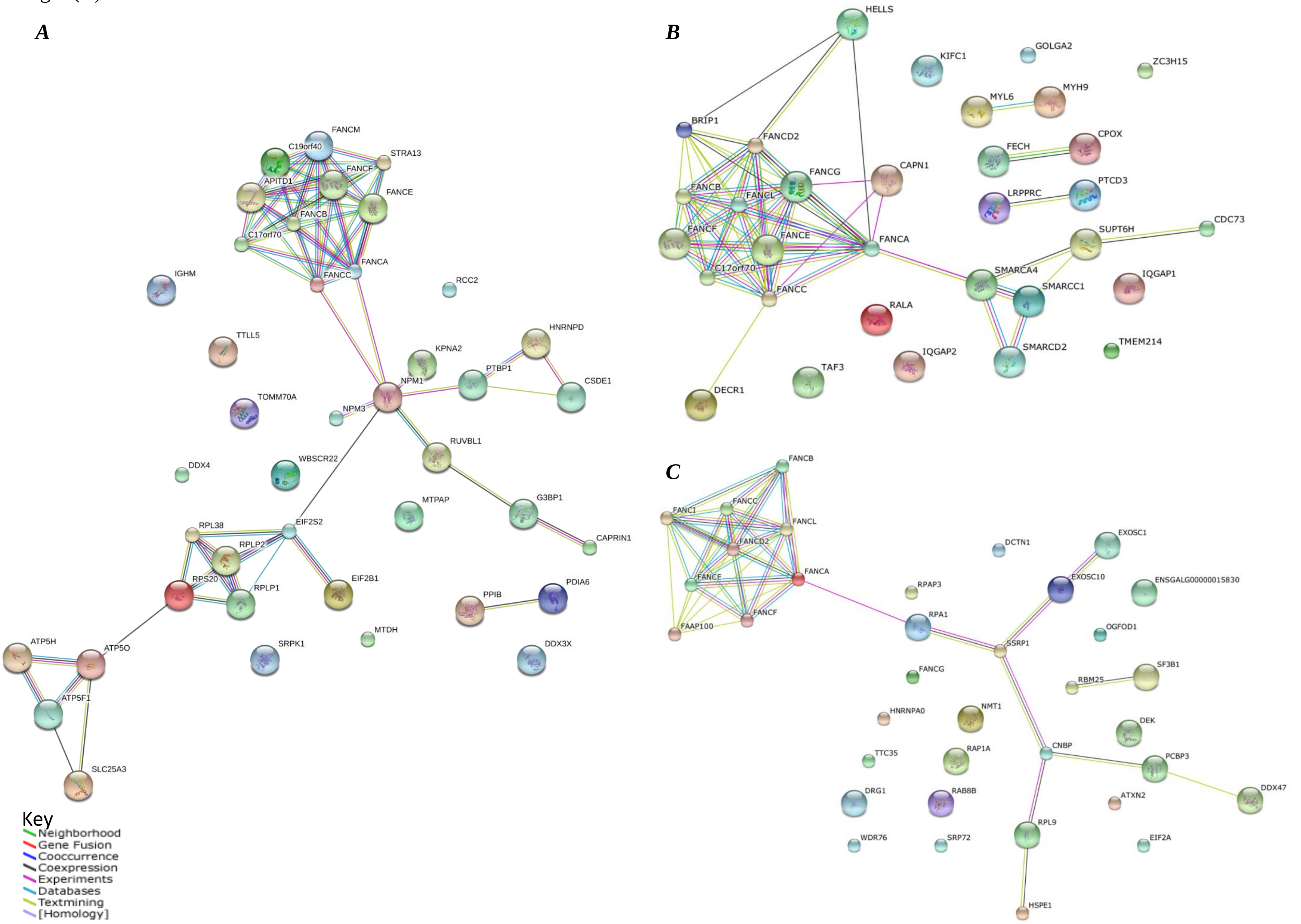

Supplement: Supplementary file 1 — Supplemental Fig. S1. Mass spectrometry data workflow and protein acceptance criteria. Supplemental Fig. S2. GO annotation and proteins over- and under-represented in DT40 lysates to determine abundance and potential contaminants. (A) Pie chart showing the GO cellular components classification of DT40. (B) Proteins under-represented in DT40 compared to the G. gallus SwissProt proteome. (C) Proteins over-represented in DT40 compared to the G. gallus SwissProt proteome. Supplemental Fig. S3. Non-specific binding proteins associated with ≥ 2 of 4 affinity resins: FLAG, TALON, Calmodulin and IgG sepharose. (A) Pie chart showing GO_Cellular component annotation. (B) GO_Molecular function annotation and (C) Kegg pathway analysis. ‘Other’ represents proteins other categories with single protein entries. Data analysed using ProteinCenter. (D) Overlap of proteins identified as the abundome and beadome. Supplemental Fig. S4. PI5P4K2α and β alignment. PI5P4K2α has unique swissprot (spQ5F356) and trembl (trF1NKL5) accessions, both included in UniprotKB G. gallus database 2012. Highlighted in purple and blue are unique peptides to PI5P4K2 α and β respectively, and in yellow are conserved peptides identified in replicate SILAC–iPAC screenings. Green are positively charged residues where K and L indicate ends of peptides that can be quantified. Supplemental Fig. S5. A.Fanconi anaemia complementation group C G. gallus UniprotKB accession alignment with peptides identified in both Calmodulin and IgG pull-downs replicate SILAC–iPAC screenings highlighted in green and IgG only in orange. Supplemental Fig. S6. Expanded PIP4Kin2 STRING network (+ 10) to identify potential missing interactors in our lists. Supplemental Fig. S7. All TALON interactors based on experimental evidence only. Supplemental Fig. S8. All FLAG interactors based on experimental evidence only. Supplemental Fig. S9. Proteins interacting with FA proteins using human accessions (A), Gallus accessions specific to only Ca [file mmc1.pdf]
