# Supplementary material for: SILAC-iPAC: A quantitative method for distinguishing genuine from non-specific components of protein complexes by parallel affinity capture
Source: J Proteomics. 2015 Feb 6;115:143–56. doi: 10.1016/j.jprot.2014.12.006 (PMC4329988; doi:10.1016/j.jprot.2014.12.006)
Supplement: Supplemental Table S1 — (A) The top 150 abundant proteins from 3 replicate DT40 lysate analyses, ranked by emPAI score. (B) The top 150 DT40 proteins identified that bind non-specifically to the resins, FLAG, TALON, Calmodulin and IgG, used in this study. [file mmc2.pdf]

**Supplemental Table S1*A*** . Top 150 abundant DT40 proteins from 3 LC-MS/MS runs ranked by calculated emPAI score.

| UNIPROTKB | GENE | MASCOT SCORE AVERAGE | EMPAI AVERAGE | EMPAI CALC AVERAGE | UNIQUE PEPTIDES | UNIQUE PEPTIDE SEQS | % SEQUENCE COVERAGE | DESCRIPTION |
|---|---|---|---|---|---|---|---|---|
| O42283 | HSPE1 | 263 | 2.87 | 9.00 | 10 | 10 | 85.29 | Heat shock protein 10 |
| G1K342 | TUBB2C | 2613 | 7.13 | 7.91 | 33 | 21 | 66.29 | Uncharacterized protein |
| P09244 | TUBB | 2992 | 8.32 | 7.91 | 33 | 22 | 72.52 | Tubulin beta-7 chain |
| Q5ZLN1 | PGAM1 | 1267 | 4.66 | 7.25 | 14 | 12 | 71.26 | Phosphoglycerate mutase 1 |
| Q6EE57 | RPS8 | 408 | 1.69 | 6.94 | 12 | 12 | 60.32 | 40S ribosomal protein S8 |
| P53478 | ACTG1 | 2139 | 10.18 | 6.74 | 29 | 22 | 66.22 | Actin, cytoplasmic type 5 |
| Q90705 | EEF2 | 2925 | 3.21 | 6.74 | 56 | 50 | 66.55 | Elongation factor 2 |
| P51913 | ENO1 | 7909 | 8.12 | 6.59 | 29 | 26 | 63.82 | Alpha-enolase |
| B4ZAD9 | EEF2 | 530 | 2.73 | 6.20 | 17 | 14 | 72.34 | Eukaryotic translation elongation factor 2 |
| P11501 | HSP90AA1 | 3043 | 4.35 | 5.81 | 51 | 45 | 57.01 | Heat shock protein HSP 90-alpha |
| P00356 | GAPDH | 1741 | 5.37 | 5.49 | 25 | 16 | 59.76 | glyceraldehyde-3-phosphate dehydrogenase |
| Q9IAY5 | SDOS | 609 | 2.06 | 5.49 | 13 | 13 | 45.00 | protein syndesmos precursor |
| Q5ZLQ6 | YWHAB | 989 | 2.86 | 5.31 | 15 | 14 | 59.84 | 14-3-3 protein beta/alpha |
| P00340 | LDHA | 2790 | 8.44 | 4.93 | 29 | 21 | 78.61 | L-lactate dehydrogenase A chain |
| Q5ZL72 | HSPD1 | 2410 | 4.36 | 4.74 | 33 | 27 | 57.77 | 60 kDa heat shock protein, mitochondrial |
| O57535 | NME2 | 531 | 9.02 | 4.62 | 11 | 9 | 74.51 | Nucleoside diphosphate kinase |
| P09203 | TUB1C | 2232 | 4.92 | 4.62 | 26 | 17 | 54.38 | Tubulin beta-1 chain |
| Q6EE60 | RPL18 | 281 | 1.68 | 4.62 | 7 | 7 | 46.39 | 60S ribosomal protein L18 |
| Q98TF8 | RPL22 | 271 | 0.51 | 4.62 | 4 | 4 | 26.56 | 60S ribosomal protein L22 |
| P00337 | LDHB | 946 | 2.65 | 4.46 | 17 | 17 | 61.56 | L-lactate dehydrogenase B chain |
| P42558 | RAN | 296 | 1.45 | 4.34 | 8 | 8 | 42.59 | GTP-binding nuclear protein Ran |
| A3RL83 | ANP32B | 292 | 0.97 | 4.01 | 9 | 9 | 30.38 | Lectin-associated matrix protein |
| E1C2A1 | ANP32A | 274 | 0.98 | 4.01 | 9 | 9 | 36.30 | Acidic leucine-rich nuclear phosphoprotein 32 family member |
| O73885 | HSPA8 | 2295 | 3.24 | 3.98 | 33 | 30 | 52.79 | Heat shock cognate 71 kDa protein |
| Q5ZKC9 | YWHAZ | 1100 | 2.86 | 3.87 | 15 | 14 | 53.06 | 14-3-3 protein zeta |
| P63247 | GNB2L1 | 690 | 1.44 | 3.83 | 15 | 15 | 47.95 | Guanine nucleotide-binding protein subunit beta-2-like 1 |
| Q5ZMN3 | PHB2 | 438 | 0.94 | 3.81 | 19 | 18 | 57.81 | Prohibitin-2 |
| P00940 | TPI1 | 465 | 2.22 | 3.64 | 11 | 11 | 64.11 | Triosephosphate isomerase |
| P04210 | L1CHV | 266 | 1.27 | 3.64 | 4 | 4 | 54.72 | Ig lambda chain V-1 region |
| Q5ZKK8 | RPL19 | 26 | 0.14 | 3.64 | 8 | 8 | 35.20 | Ribosomal protein L19 |
| Q5ZLC5 | ATP5B | 1662 | 2.67 | 3.64 | 28 | 22 | 59.29 | ATP synthase subunit beta, mitochondrial |
| Q5ZMD1 | YWHAQ | 537 | 1.74 | 3.64 | 12 | 11 | 44.49 | 14-3-3 protein theta |
| Q5ZMT0 | YWHAE | 837 | 3.05 | 3.64 | 15 | 14 | 48.63 | 14-3-3 protein epsilon |
| Q90ZG0 | FKBP1A | 42 | 0.66 | 3.64 | 2 | 2 | 25.00 | Peptidyl-prolyl cis-trans isomerase |
| Q5ZJU3 | ASNS | 885 | 1.10 | 3.52 | 20 | 19 | 40.29 | Asparagine synthetase [glutamine-hydrolyzing] |
| Q8JFP1 | EIF4A2 | 857 | 1.99 | 3.49 | 19 | 19 | 47.67 | Eukaryotic initiation factor 4A-II |
| F2Z4K7 | RPS3A | 143 | 1.08 | 3.44 | 15 | 14 | 50.76 | Uncharacterized protein |
| Q90835 | EEF1A1 | 799 | 2.12 | 3.44 | 16 | 15 | 38.96 | Elongation factor 1-alpha 1 |
| P47836 | RPS4X | 373 | 3.39 | 3.28 | 17 | 16 | 53.23 | 40S ribosomal protein S4 |
| Q5ZJC1 | RPS3 | 358 | 1.26 | 3.28 | 13 | 13 | 54.51 | Putative uncharacterized protein |
| P79781 | RPS27A | 235 | 0.97 | 3.22 | 7 | 6 | 36.54 | Ubiquitin-40S ribosomal protein S27a |
| Q90694 | CDC42 | 80 | 1.37 | 3.22 | 5 | 5 | 32.46 | Cell division control protein 42 homolog |
| F1N9J7 | TUBA3E | 1164 | 1.74 | 3.16 | 17 | 15 | 41.78 | PREDICTED: tubulin alpha-1C chain |
| Q9I9V6 | GAG | 193 | 0.47 | 3.12 | 8 | 7 | 26.42 | Gag polyprotein |
| P00548 | PKM2 | 2455 | 3.64 | 3.08 | 30 | 24 | 56.79 | Pyruvate kinase muscle isozyme |
| P47826 | RPLP0 | 998 | 2.02 | 3.08 | 11 | 11 | 49.68 | 60S acidic ribosomal protein P0 |
| P68034 | ACTC1 | 927 | 3.18 | 3.08 | 19 | 15 | 35.81 | Actin, alpha cardiac muscle 1 |
| F1P1A5 | TKT | 735 | 1.20 | 2.98 | 22 | 21 | 36.36 | PREDICTED: transketolase |

| | | | | | | | | |
|---|---|---|---|---|---|---|---|---|
| P02552 | TUBA1C | 806 | 2.47 | 2.98 | 6 | 6 | 40.56 | Tubulin alpha-1 chain |
| Q5ZJ56 | RPL7 | 66 | 0.93 | 2.98 | 11 | 9 | 38.62 | 60S ribosomal protein L7 |
| Q9I9V5 | GAG | 258 | 2.08 | 2.98 | 8 | 7 | 33.89 | Gag polyprotein |
| Q9I9V7 | GAG | 215 | 1.39 | 2.98 | 8 | 7 | 33.89 | Gag polyprotein |
| D5M8S3 | PHB | 350 | 0.88 | 2.87 | 11 | 11 | 52.94 | Prohibitin transcript variant 2 |
| P15771 | NCL | 1184 | 1.15 | 2.86 | 28 | 25 | 35.73 | nucleolin/C23 |
| D0EKR3 | PPIA | 528 | 5.56 | 2.83 | 13 | 9 | 44.24 | Peptidyl-prolyl cis-trans isomerase |
| F1NGS7 | ARF4 | 94 | 0.83 | 2.83 | 7 | 7 | 40.56 | Uncharacterized protein |
| P09653 | TUBB6 | 938 | 1.14 | 2.79 | 19 | 12 | 33.41 | Tubulin beta-5 chain |
| Q5F424 | CCT2 | 1070 | 1.57 | 2.76 | 26 | 22 | 52.52 | Uncharacterized protein |
| O93466 | RAC3 | 104 | 0.33 | 2.73 | 6 | 6 | 26.04 | GTPase cRac1B |
| Q6EE31 | CCT8 | 710 | 1.91 | 2.73 | 28 | 24 | 46.53 | T-complex protein 1 subunit theta |
| Q9W7I5 | RBBP4 | 157 | 0.31 | 2.73 | 8 | 8 | 19.29 | Histone-binding protein RBBP4 |
| P38024 | PAICS | 747 | 0.95 | 2.67 | 18 | 15 | 45.77 | Multifunctional protein ADE2 |
| Q5ZJK8 | CCT7 | 1323 | 1.45 | 2.65 | 23 | 20 | 45.03 | T-complex protein 1 subunit eta |
| P51903 | PGK1 | 774 | 2.57 | 2.63 | 19 | 17 | 47.00 | Phosphoglycerate kinase |
| P20763 | | 134 | 0.50 | 2.59 | 5 | 5 | 40.37 | Ig lambda chain C region |
| Q6ITC7 | RPS13 | 44 | 0.43 | 2.59 | 5 | 5 | 39.74 | 40S ribosomal protein S13 |
| Q6SVA6 | RPSA | 269 | 0.62 | 2.59 | 7 | 7 | 33.77 | DMRT1 isoform e |
| P09652 | TUBB3 | 1518 | 2.10 | 2.55 | 20 | 12 | 32.96 | Tubulin beta-4 chain |
| Q6JAY6 | GAG | 197 | 1.55 | 2.51 | 7 | 7 | 26.27 | Gag polyprotein |
| Q9PTD6 | RPS6 | 105 | 0.56 | 2.51 | 8 | 7 | 35.22 | 40S ribosomal protein S6 |
| Q9I9V4 | GAG | 379 | 1.54 | 2.46 | 12 | 9 | 30.83 | Gag polyprotein |
| Q90679 | THYN1 | 103 | 0.75 | 2.38 | 9 | 9 | 43.80 | Thymocyte nuclear protein 1 |
| Q9DEA3 | PCNA | 194 | 1.37 | 2.38 | 12 | 10 | 45.80 | Proliferating cell nuclear antigen |
| Q8UVX3 | ATP5A1 | 1216 | 1.22 | 2.27 | 17 | 17 | 38.34 | ATP synthase subunit alpha |
| Q5ZL53 | PABPC1 | 422 | 0.80 | 2.27 | 21 | 20 | 35.32 | Putative uncharacterized protein |
| Q04619 | HSP90AB1 | 966 | 0.92 | 2.26 | 27 | 26 | 33.38 | Heat shock cognate protein HSP 90-beta |
| F1NG87 | TTC28 | 24 | | 2.16 | 3 | 3 | 26.09 | similar to OTTHUMP00000028696 |
| F1P3F1 | AHCY | 456 | 0.70 | 2.16 | 14 | 13 | 39.26 | adenosylhomocysteinase |
| H9KZC8 | PA2G4 | 191 | 1.09 | 2.16 | 13 | 13 | 42.27 | similar to proliferation-associated protein 1, partial |
| O42388 | UBA52 | 129 | 0.51 | 2.16 | 5 | 4 | 25.78 | Ubiquitin-ribosomal protein fusion protein |
| P16039 | NPM1 | 224 | 0.62 | 2.16 | 11 | 11 | 34.01 | Nucleophosmin |
| P18359 | DSTN | 354 | 2.72 | 2.16 | 7 | 6 | 44.85 | Destrin |
| P18660 | RPLP1 | 63 | 0.30 | 2.16 | 1 | 1 | 14.04 | 60S acidic ribosomal protein P1 |
| P53449 | ALDOC | 486 | 1.86 | 2.16 | 4 | 3 | 35.04 | Fructose-bisphosphate aldolase C |
| Q02960 | MIF | 107 | 1.05 | 2.16 | 5 | 4 | 34.78 | Macrophage migration inhibitory factor |
| Q5F3R9 | SET | 136 | 0.48 | 2.16 | 5 | 5 | 28.16 | Putative uncharacterized protein |
| Q5ZHW8 | RPS14 | 200 | 0.45 | 2.16 | 4 | 4 | 28.48 | Uncharacterized protein |
| Q5ZL59 | UBE2D3 | 59 | 0.20 | 2.16 | 2 | 2 | 23.81 | Uncharacterized protein |
| Q6EE59 | RPL4 | 184 | 0.64 | 2.16 | 8 | 8 | 35.36 | Ribosomal protein L4 |
| Q6EE62 | RPL10A | 66 | 0.35 | 2.16 | 5 | 5 | 28.80 | Ribosomal protein |
| Q6JAX8 | GAG | 163 | 1.26 | 2.16 | 7 | 7 | 26.27 | Gag polyprotein |
| P09102 | P4HB, PDI, PDIA1 | 395 | 0.39 | 2.05 | 18 | 18 | 38.21 | protein disulfide isomerase |
| Q5F411 | CCT5 | 678 | 0.53 | 2.05 | 19 | 18 | 32.72 | Uncharacterized protein |
| F6R1X6 | SSB | 205 | 0.41 | 2.03 | 18 | 18 | 43.56 | Sjogren syndrome antigen B (autoantigen La) isoform 1 |
| Q5ZIQ3 | HNRPK | 203 | 0.22 | 2.02 | 11 | 11 | 35.83 | Heterogeneous nuclear ribonucleoprotein K |
| F1NGA2 | ATP5A1W | 843 | 1.12 | 2.01 | 11 | 11 | 32.40 | ATP synthase alpha subunit |
| P08110 | HSP90B1, TRA1 | 449 | 0.32 | 1.99 | 22 | 22 | 26.67 | Endoplasmin |
| Q5F470 | RAB8A | 33 | 0.30 | 1.93 | 7 | 7 | 40.10 | Ras-related protein Rab-8A |
| Q9W744 | DDX5 | 270 | 0.27 | 1.93 | 17 | 16 | 27.39 | DEAD-box RNA helicase |
| Q03853 | SE21Q1b | 649 | 0.98 | 1.91 | 19 | 15 | 28.67 | Gag protein |
| Q8UWG7 | RPL6 | 84 | 0.32 | 1.89 | 7 | 7 | 22.82 | 60S ribosomal protein L6 |
| Q9I9V2 | GAG | 365 | 1.18 | 1.89 | 10 | 7 | 26.94 | Gag polyprotein |
| Q5F4A4 | IMPDH2 | 438 | 0.67 | 1.87 | 17 | 17 | 37.55 | Inosine-5'-monophosphate dehydrogenase |
| H9L011 | LOC426023 | 126 | 0.57 | 1.85 | 5 | 5 | 32.12 | nuclear poly(C)-binding protein, splicevariant E |

| | | | | | | | | |
|---|---|---|---|---|---|---|---|---|
| F1NXW3 | RPS15A | 127 | 1.78 | 1.85 | 8 | 7 | 42.31 | 40S ribosomal protein S15a isoform 1 |
| P41125 | RPL13 | 126 | 0.67 | 1.85 | 7 | 7 | 28.44 | 60S ribosomal protein L13 |
| Q5ZKJ3 | DUT | 134 | 0.68 | 1.85 | 5 | 5 | 30.72 | Uncharacterized protein |
| Q6JAY3 | GAG | 220 | 1.55 | 1.85 | 8 | 7 | 33.86 | Gag polyprotein |
| P09207 | TUBB1 | 628 | 0.76 | 1.82 | 15 | 10 | 24.44 | Tubulin beta-6 chain |
| Q03852 | RAV-0 | 703 | 1.25 | 1.81 | 19 | 15 | 29.10 | Gag protein |
| P51417 | RPL15 | 74 | 0.90 | 1.78 | 6 | 6 | 42.42 | 60S ribosomal protein L15 |
| Q5F4A3 | ANP32E | 113 | 0.54 | 1.78 | 4 | 4 | 19.92 | Acidic leucine-rich nuclear phosphoprotein 32 family member |
| Q5ZJ54 | CCT6A | 852 | 0.64 | 1.78 | 14 | 13 | 31.51 | T-complex protein 1 subunit zeta |
| Q5ZME2 | MDH1 | 91 | 0.19 | 1.78 | 10 | 9 | 30.54 | Malate dehydrogenase, cytoplasmic |
| Q9DDH7 | ATP5A1 | 217 | 1.13 | 1.78 | 4 | 4 | 39.09 | ATP synthase alpha subunit |
| O93382 | GDI2 | 746 | 0.99 | 1.75 | 15 | 13 | 39.06 | Rab-GDP dissociation inhibitor |
| H9KZF8 | ATP5C1 | 50 | 0.47 | 1.68 | 6 | 6 | 22.87 | similar to ATP synthase, H+ transporting, mitochondrial F1 co |
| P00508 | GOT2 | 494 | 0.60 | 1.68 | 16 | 16 | 37.59 | Aspartate aminotransferase, mitochondrial |
| Q6EE30 | EEF1G | 401 | 0.89 | 1.68 | 11 | 11 | 24.77 | Eukaryotic translation elongation factor 1 |
| Q5ZMU3 | GPI | 712 | 0.85 | 1.65 | 18 | 16 | 28.93 | Glucose-6-phosphate isomerase |
| Q5ZIX2 | ETFA | 191 | 0.29 | 1.64 | 8 | 8 | 30.92 | Putative uncharacterized protein |
| P49702 | ARF5 | 103 | 0.58 | 1.61 | 5 | 5 | 31.11 | ADP-ribosylation factor 5 |
| Q5ZJX9 | PSMA5 | 29 | 0.42 | 1.61 | 5 | 5 | 23.24 | Proteasome subunit alpha type |
| Q5ZMT1 | ARHGDIA | 152 | 0.71 | 1.61 | 5 | 4 | 38.24 | Putative uncharacterized protein |
| Q90WA6 | HSP90B1 | 449 | 0.33 | 1.60 | 20 | 20 | 24.03 | Heat shock protein 108 |
| E1C4M0 | RPS2 | 290 | 1.64 | 1.51 | 10 | 10 | 29.53 | 40S ribosomal protein S2 |
| H9L366 | IDH2 | 45 | 0.42 | 1.51 | 2 | 2 | 50.67 | hypothetical protein LOC431056 |
| D0VX32 | UQCRQ | 40 | 0.35 | 1.51 | 2 | 2 | 28.24 | Mitochondrial ubiquinol-cytochrome c reductase ubiquinone- |
| P09643 | TUBA4A | 2112 | 1.60 | 1.51 | 9 | 7 | 25.16 | Tubulin alpha-4 chain |
| Q5ZI56 | RPL11 | 199 | 0.52 | 1.51 | 5 | 5 | 26.53 | Putative uncharacterized protein |
| F1NSQ1 | AIMP2 | 82 | 0.11 | 1.42 | 6 | 6 | 29.89 | multisynthetase complex p38 auxiliary component |
| E1BYW9 | PSMB3 | 243 | 0.14 | 1.37 | 3 | 3 | 20.00 | Proteasome subunit beta type |
| H9L132 | LOC772076 | 35 | | 1.37 | 3 | 3 | 24.86 | Uncharacterized protein |
| F1NJF0 | C1QBP | 71 | 0.25 | 1.37 | 3 | 3 | 17.55 | p32 subunit of splicing factor SF2 |
| P31335 | ATIC | 212 | 0.34 | 1.37 | 14 | 14 | 30.02 | Bifunctional purine biosynthesis protein PURH |
| P43347 | TPT1 | 52 | 0.61 | 1.37 | 4 | 3 | 17.44 | Translationally-controlled tumor protein homolog |
| Q5F3J5 | PSME3 | 28 | 0.24 | 1.37 | 7 | 7 | 26.38 | Proteasome activator complex subunit 3 |
| Q5F3W6 | YWHAG | 228 | 0.56 | 1.37 | 8 | 7 | 21.86 | 14-3-3 protein gamma |
| Q5ZKJ2 | YWHAH | 228 | 0.56 | 1.37 | 8 | 7 | 21.05 | Uncharacterized protein |
| Q90593 | HSPA5 | 580 | 0.49 | 1.35 | 16 | 16 | 27.15 | 78 kDa glucose-regulated protein |
| Q5ZL82 | IDH2 | 156 | 0.46 | 1.35 | 12 | 12 | 25.44 | Isocitrate dehydrogenase [NADP] |
| F1P582 | UQCRC2 | 283 | 0.30 | 1.34 | 8 | 8 | 20.35 | Uncharacterized protein |
| Q5F491 | DDX3X | 620 | 0.70 | 1.34 | 14 | 14 | 25.96 | Putative uncharacterized protein |
| Q5ZM75 | SARS | 301 | 0.46 | 1.29 | 13 | 13 | 27.63 | Putative uncharacterized protein |
| Q6WNG8 | HNRNPH1 | 345 | 0.25 | 1.29 | 9 | 9 | 21.00 | Heterogeneous nuclear ribonucleoprotein H1-like protein |
| Q5ZLI2 | PSMA3 | 93 | 0.12 | 1.28 | 5 | 5 | 19.61 | Proteasome subunit alpha type |
| Q90602 | HNRNPAB | 60 | 0.22 | 1.28 | 6 | 6 | 20.20 | Single stranded D box binding factor |
| Q90626 | HNRNPAB | 46 | 0.10 | 1.28 | 6 | 6 | 23.51 | Ribonucleoprotein |
| Q5ZLG0 | AACS | 113 | 0.14 | 1.21 | 12 | 12 | 14.54 | Acetoacetyl-CoA synthetase |
| Q8JG64 | PDIA3 | 196 | 0.57 | 1.21 | 11 | 11 | 22.18 | Protein disulfide-isomerase A3 |

Key

emPAI: Exponentially Modified Protein Abundance Index (imported from MASCOT)

emPAI_Calc. Calculated emPAI considering peptides with masses between 700 and 2800 Da.
