# Supplementary material for: SILAC-iPAC: A quantitative method for distinguishing genuine from non-specific components of protein complexes by parallel affinity capture
Source: J Proteomics. 2015 Feb 6;115:143–56. doi: 10.1016/j.jprot.2014.12.006 (PMC4329988; doi:10.1016/j.jprot.2014.12.006)
Supplement: Supplemental Table S1 — (A) The top 150 abundant proteins from 3 replicate DT40 lysate analyses, ranked by emPAI score. (B) The top 150 DT40 proteins identified that bind non-specifically to the resins, FLAG, TALON, Calmodulin and IgG, used in this study. [file mmc3.pdf]

Supplemental Table S1*B* . Proteins identified in wt DT40 affinity purifications using FLAG, TALON, Calmodulin and IgG sepharose affinity resins.

ID	Protein Description	Affinity resin				GO Annotation			Mascot score				Unique peptides			
		FLAG	TALON	Cal	IgG	Cell component	Bio. Process	Mol.	FLAG	TALON	Cal	IgG	FLAG	TALON	Cal	IgG
Q90593	78 kDa glucose-regulated protein	1	1	1	1	cs,mb,er,cp,ol,c	cx,cob,mp,reg,r	protein	2332	610	34	23	34	13	5	3
P08106	Heat shock 70 kDa protein	1	1	1	1	cs	cob,res	protein	909	523	34	23	15	12	6	4
P51913	Alpha-enolase	1	1	1	1	cp,cl	mp	metal ion	614	1115	654	136	12	17	13	10
Q90835	Elongation factor 1-alpha 1	1	1	1	1	cp	mp	protein	341	503	40	205	14	11	8	9
Q6PVZ3	Type II alpha-keratin IIC	1	1	1	1	ck		structural	298	373	361	378	13	8	11	10
Q6PVZ5	Type II alpha-keratin IIA	1	1	1	1	ck		motor	270	320	272	298	13	9	9	7
O93532	Keratin, type II cytoskeletal cochleal	1	1	1	1	ck		structural	264	340	275	317	11	11	8	11
P00340	L-lactate dehydrogenase A chain	1	1	1	1	cp,cl	mp	nucleotide	262	376	144	314	4	7	3	5
Q5ZHW8	Uncharacterized protein	1	1	1	1	mt,cp,rb,ol,cl,nu	mp,reg	RNA	212	200	48	82	5	3	1	3
P00548	Pyruvate kinase muscle isozyme	1	1	1	1	cp,cl	mp	metal ion	204	271	129	179	5	10	10	6
O93256	Keratin, type I cytoskeletal 19	1	1	1	1	ck		motor	164	147	195	70	8	6	5	2
E1BY89	Uncharacterized protein	1	1	1	1	cp,rb,ol,cl,nu	mp	structural	121	132	43	41	3	3	3	3
P0C1H4	Histone H2B 5	1	1	1	1	ch,nu	cob,mp	DNA	120	144	320	720	3	6	6	8
Q6PVZ4	Type II alpha-keratin IIB	1	1	1	1	ck		motor	118	225	209	215	8	5	8	5
H9KZP2	similar to cytokeratin 8	1	1	1	1	ck		motor	114	171	160	163	6	4	5	4
P00356	glyceraldehyde-3-phosphate	1	1	1	1		mp	catalytic	92	343	41	141	7	7	5	8
P07322	Beta-enolase	1	1	1	1	cp,cl	mp	metal ion	85	166	211	80	1	2	2	3
Q5ZLN1	Phosphoglycerate mutase 1	1	1	1	1	cp,cl	mp	catalytic	71	43	61	56	3	2	2	2
Q6PVZ2	Type I alpha-keratin 15	1	1	1	1	ck		structural	60	51	51	40	8	6	12	5
F1NDP0	Keratin, type I cytoskeletal 14	1	1	1	1	ck		structural	53	47	39	40	5	8	4	2
P62801	Histone H4	1	1	1	1	ch,nu	dev,cob,mp,reg	DNA	52	177	278	482	5	5	6	6
Q5ZMD6	Histone H2A.Z	1	1	1	1	ch,nu	cob,mp	DNA	39	50	69	106	5	2	3	4
D0EKR3	Peptidyl-prolyl cis-trans isomerase	1	1	1	1		mp	catalytic	36	136	113	120	3	6	6	6
P60706	Actin, cpic 1	1		1	1	ck,cp		nucleotide	1214		237	460	16	0	10	13
P09203	Tubulin beta-1 chain	1	1		1	ck,cp	cob,mp,ccm	nucleotide	761	505		40	16	11	0	3
P02552	Tubulin alpha-1C chain	1	1		1		cob,mp	nucleotide	749	268		34	7	8	0	3
P08070	Tubulin alpha-2 chain	1	1	1		ck,cp	cob,mp,ccm	nucleotide	657	150	32		9	6	1	0
P68034	Actin, alpha cardiac muscle 1	1	1	1		ck,cp	cx,cob,mp,ccm	protein	450	571	200		10	13	8	0
Q8UWG7	60S ribosomal protein L6	1	1		1	cp,rb,cl	mp	structural	418	136		23	11	9	0	2
P09653	Tubulin beta-5 chain	1	1		1	ck,cp	cob,mp,ccm	nucleotide	395	138		40	12	7	0	3
Q6EE60	60S ribosomal protein L18	1	1	1		cp,rb	mp	structural	350	328	36		9	5	4	0
O57391	Gamma-enolase	1	1	1		cp,cl	mp	metal ion	238	295	275		3	5	4	0
Q5ZL72	60 kDa heat shock protein,	1	1	1		ex,cs,mb,mt,cp,	cx,cp,mp,reg,re	protein	209	281	26		6	12	3	0
Q8JFP1	Eukaryotic initiation factor 4A-II	1	1		1		mp	RNA	178	172		74	5	5	0	4
P61355	60S ribosomal protein L27	1	1		1	cp,rb,cl	mp	structural	141	41		34	5	3	0	1
Q98TF8	60S ribosomal protein L22	1	1	1		cp,rb	mp	structural	139	193	56		3	3	2	0
Q5ZLC5	ATP synthase subunit beta,	1	1		1	mb,mt,cp	dev,tp,mp,reg,	tper	129	277		62	7	9	0	3

P63247	Guanine nucleotide-binding protein	1	1		1	mb,cp,nu	cx,dev,cob,tp,	protein	120	43		29	4	6	0	4
Q03853	Gag protein	1	1	1			mp	metal ion	63	305	90		12	9	6	0
Q5ZKC9	14-3-3 protein zeta	1	1	1		mt,cp,nu	tp	protein	48	107	35		3	5	2	0
P14732	Lamin-B2	1	1		1	ck,mb,nu	mp,reg	motor	46	32		37	5	9	0	5
Q6ITC7	40S ribosomal protein S13	1	1	1		cp,rb,ol,nu,cl	mp,reg	RNA	43	75	23		3	2	1	0
P09654	Vimentin	1	1	1		ck,mb,cp	cob,mp,reg	protein	39	47	48		6	9	5	0
E1BU66	Uncharacterized protein	1	1		1	cp,rb,cl	mp	structural	37	62		39	3	4	0	1
F6R1X6	Sjogren syndrome antigen B	1	1		1				36	105		47	6	8	0	4
Q5F411	Uncharacterized protein	1	1		1	ck,cp,ol,nu,cl	mp	protein	34	92		42	1	9	0	3
P51903	Phosphoglycerate kinase	1	1		1	cp,ch,cl	mp	nucleotide	31	74		28	3	8	0	5
F1NF88	phosphatase and actin regulator 3	1	1		1				26	32		29	3	5	0	3
P11533	Dystrophin	1	1		1	ck,mb,cp		protein	24	24		24	14	10	0	12
Q5ZLX5	similar to RAN-binding protein 2-like	1	1	1			tp		23	41	25		2	4	2	0
F1N9H4	elongation factor 1 alpha 2		1	1	1			nucleotide		419	40	158	0	7	6	7
Q03852	Gag protein		1	1	1		mp	metal ion		302	90	35	0	8	6	2
Q6PVZ1	Keratin 14		1	1	1					63	98	70	0	2	4	2
O42283	Heat shock protein 10		1	1	1	mt,cp	mp,res	nucleotide		48	135	44	0	2	2	3
P08287	Histone H1.11L		1	1	1	ch,nu	cob,mp	DNA		47	54	60	0	3	2	3
Q5ZMJ7	protein CIP2A homolog		1	1	1	ck,mb,cp,ol,ch,[illegible]	cob,tp,rep	protein		35	28	39	0	2	3	3
2.3E+08	heat shock 105kDa	1	1						4329	867			51	15	0	0
Q5ZK62	Arf-GAP with coiled-coil, ANK repeat	1	1				mp,reg	protein	2750	186			39	6	0	0
Q5ZMU9	Valosin containing protein	1	1				mp,res,rep	protein	1760	309			36	9	0	0
O73885	Heat shock cognate 71 kDa protein	1	1			cs,cp	cob,tp,res	nucleotide	1739	610			24	18	0	0
Q5ZL26	Phosphoribosyl pyrophosphate	1	1				mp	metal ion	1557	230			21	1	0	0
Q5F4A4	Inosine-5'-monophosphate	1	1				mp	protein	1501	249			23	12	0	0
P09244	Tubulin beta-7 chain	1	1			ck,cp	cob,mp,ccm	nucleotide	1251	619			18	12	0	0
P15771	Nucleolin	1	1			ol,nu		DNA	1059	541			21	18	0	0
Q7S163	Heat shock protein 70	1		1		cs,mb,mt,cp,ol,	cob,mp,reg,res,	protein	909		34		14	0	6	0
E1BYN9	phosphoribosyl pyrophosphate	1	1				mp	metal ion	720	184			10	4	0	0
D6PVB7	Heat shock protein 70	1	1				cob,res	nucleotide	707	116			14	9	0	0
Q9IAY5	protein syndesmos	1	1						699	56			11	5	0	0
P09652	Tubulin beta-4 chain	1	1			ck,cp	cob,mp,ccm	nucleotide	679	313			13	10	0	0
Q5ZII4	Putative uncharacterized protein	1	1				mp	metal ion	618	68			9	2	0	0
P47826	60S acidic ribosomal protein P0	1	1			cp,rb	mp	structural	611	434			9	10	0	0
F2Z4K7	Uncharacterized protein	1	1			cp,rb,ol,cl,nu	cx,mp,reg	structural	408	426			11	14	0	0
Q5F491	Putative uncharacterized protein	1	1					nucleotide	383	201			10	6	0	0
E1C4M0	40S ribosomal protein S2	1	1			cp,rb	mp	RNA	380	199			9	9	0	0
P41125	60S ribosomal protein L13	1	1			cp,rb,cl	mp	structural	369	330			5	6	0	0
Q5ZII1	Uncharacterized protein	1	1			cp,rb,ol,nu,cl	mp	structural	364	79			15	9	0	0
Q5ZJC1	Putative uncharacterized protein	1	1			cp,rb	mp	RNA	363	334			9	7	0	0

P09207	Tubulin beta-6 chain	1	1			ck,cp	cob,mp,ccm	nucleotide	358	149			9	8	0	0
Q6EE57	40S ribosomal protein S8	1	1			cp,rb	mp	structural	328	133			7	5	0	0
Q5ZI49	Ribose-phosphate	1	1				mp	protein	308	39			8	3	0	0
Q5ZIG4	Putative uncharacterized protein	1	1				mp	nucleotide	297	65			6	1	0	0
Q5ZLG7	Putative uncharacterized protein	1	1			mb,mt,cp	tp	tper	285	187			14	8	0	0
Q5ZME1	Uncharacterized protein	1	1			sc,ol,nu	mp,tp,reg	DNA	234	206			5	3	0	0
Q5ZJZ2	Putative uncharacterized protein	1	1			cp,rb	mp	structural	232	31			11	7	0	0
Q5ZMJ6	Uncharacterized protein	1	1			mt,mb,cp	cx,cob,tp,reg	tper	227	188			10	5	0	0
Q5ZLE6	Eukaryotic translation initiation	1	1			cp	mp	protein	213	93			8	4	0	0
P32429	60S ribosomal protein L7a	1	1			cp,rb			201	96			14	6	0	0
Q08200	60S ribosomal protein L10	1	1			cp,rb,nu	mp	structural	200	86			8	2	0	0
Q9W744	DEAD-bo1 RNA helicase	1	1			nu		nucleotide	195	61			14	11	0	0
F1NI22	ATP synthase alpha subunit, partial	1	1			mb	mp,tp	tper	181	238			10	7	0	0
Q9PTD6	40S ribosomal protein S6	1	1			cp,rb,ol,nu	cx,mp,reg,res,c	protein	179	299			9	9	0	0
P47836	40S ribosomal protein S4	1	1			cp,rb	mp	RNA	170	254			7	7	0	0
P67883	60S ribosomal protein L30	1	1			cp,rb,cl	mp	structural	163	117			7	4	0	0
E1BS06	Uncharacterized protein	1	1			cp,rb,nu,cl	cp,mp	nucleotide	160	117			7	4	0	0
Q5ZI56	Putative uncharacterized protein	1	1			cp,rb	mp	structural	154	148			2	3	0	0
Q5ZKK8	Ribosomal protein L19	1	1			cp,rb,cl	mp	structural	154	33			4	5	0	0
E1C8R1	Probable ATP-dependent RNA	1	1					nucleotide	152	55			4	2	0	0
Q5ZJN2	Ras-related protein Rab-11A	1	1			ck,mb,mt,cp,gg	cob,tp,mp,reg,r	protein	143	61			8	3	0	0
H9L213	Uncharacterized protein	1	1			cp,rb	mp	structural	130	114			3	5	0	0
Q5ZM98	Stress-70 protein, mitochondrial	1	1			mt,cp	cob,mp	protein	126	48			7	8	0	0
Q5ZJ56	60S ribosomal protein L7	1	1			cp,rb,cl	mp	protein	123	99			11	8	0	0
F1NTT2	ribosomal protein S28	1	1			cp,rb	mp	structural	110	168			4	2	0	0
P51417	60S ribosomal protein L15	1	1			cp,rb	mp	structural	98	50			6	3	0	0
Q90705	Elongation factor 2	1	1			cp	mp	RNA	91	148			6	14	0	0
Q9YGQ1	elongation factor 1	1	1						87	163			1	1	0	0
P11501	Heat shock protein HSP 90-alpha	1	1			cp	mp,reg,res	protein	82	503			10	17	0	0
Q5ZKA5	Bifunctional	1	1			mt,cp	mp	nucleotide	76	163			5	2	0	0
P62846	40S ribosomal protein S15a	1	1			cp,rb	mp	structural	75	92			4	4	0	0
Q98TF7	60S ribosomal protein L35	1	1			cp,rb	mp	structural	73	64			2	2	0	0
Q9PT84	voltage-gated potassium channel	1	1						72	75			6	1	0	0
Q5ZL53	Putative uncharacterized protein	1	1					RNA	66	70			9	13	0	0
E1C6T8	Uncharacterized protein	1	1				mp	protein	63	26			6	2	0	0
P04210	immunoglobulin light-chain VJ	1	1						60	59			1	1	0	0
Q04619	Heat shock cognate protein HSP 90-	1	1			cp,cl	mp,res	protein	56	116			4	5	0	0
P47832	ribosomal protein L26	1	1						50	136			7	8	0	0
P79781	Ubiquitin-40S ribosomal protein	1	1			cp,rb,ol,nu	mp,reg,res,cc,d	protein	48	110			5	5	0	0
Q98TF6	60S ribosomal protein L36	1	1			cp,rb	mp	structural	48	58			3	5	0	0

Q5ZKJ2	Uncharacterized protein	1	1			cp	tp,mp,reg,res,c	protein	46	46			2	2	0	0
Q5ZMD1	14-3-3 protein theta	1	1			ck,cp,nu	mp,tp,reg,res,c	protein	46	30			3	4	0	0
Q5ZMT0	14-3-3 protein epsilon	1	1			cp		protein	46	25			4	2	0	0
O57535	Nucleoside diphosphate kinase	1	1			mb,mt,cp	cx,cp,dev,mp,r	metal ion	44	58			4	4	0	0
H9L3K5	similar to strabismus	1	1			mb	dev		42	30			2	2	0	0
P02263	Histone H2A-IV	1		1		ch,nu	cob,mp	DNA	39		236		3	0	5	0
E1BWJ7	Uncharacterized protein	1	1					RNA	37	31			8	5	0	0
P18460	inositol 1,4,5-trisphosphate	1	1			mb	tp	tper	33	33			11	6	0	0
F1NZV1	Uncharacterized protein	1	1			mb,er,cp	tp,reg,res,cc	signal	33	33			16	11	0	0
Q5ZIH1	Uncharacterized protein	1	1				mp,reg	nucleotide	32	30			5	2	0	0
E1C903	Uncharacterized protein	1	1					RNA	31	31			4	6	0	0
Q98TH5	Ribosomal protein S11	1	1			cp,rb	mp	structural	31	27			4	4	0	0
P30352	Serine/arginine-rich splicing factor 2	1	1			nu	mp	RNA	30	43			1	3	0	0
Q5ZIB2	Fas-binding factor 1 homolog	1		1		ck,mb,cp	cob,tp,mp,reg	tper	29		48		4	0	4	0
Q5ZI60	Uncharacterized protein	1	1			ck	cob	protein	28	48			3	9	0	0
F1P2S8	A-kinase anchor protein 9	1	1			ck,mb	mp,tp,reg	tper	27	44			17	13	0	0
P00508	Aspartate aminotransferase,	1	1			mt,cp,ol	mp	protein	27	32			4	7	0	0
Q8JG64	Protein disulfide-isomerase A3	1	1			er,cp,ol	cx,mp,reg,ch	catalytic	26	317			2	16	0	0
P81021	Vigilin	1	1			cp		RNA	26	31			9	6	0	0
Q5ZJ86	Putative uncharacterized protein	1		1		cp	mp	nucleotide	26		28		12	0	5	0
Q5ZL27	Putative uncharacterized protein	1	1			cp	cob		25	160			3	1	0	0
Q5ZKU2	Putative uncharacterized protein	1			1	ch	mp,res	DNA	25			25	9	0	0	4
P00337	L-lactate dehydrogenase B chain	1	1			mt,cp,cl	mp	nucleotide	24	105			3	7	0	0
Q90679	Thymocyte nuclear protein 1	1	1			nu			24	42			5	4	0	0
Q01841	gamma-glutamyltransferase-like 3	1	1					catalytic	24	33			1	2	0	0
P53449	Fructose-bisphosphate aldolase C		1	1		cp,cl	mp	catalytic		137	42		0	3	1	0
P18359	Destrin		1		1			protein		105		27	0	6	0	1
Q5ZLQ6	14-3-3 protein beta/alpha		1	1		ck,cp,nu	mp,tp,reg	protein		91	35		0	5	2	0
P08285	Histone H1.03		1		1	ch,nu	cob,mp	DNA		47		60	0	3	0	3
Q5ZK51	gg SNAP receptor complex member		1	1		mb,cp,gg	tp			39	30		0	3	1	0
Q5F454	Uncharacterized protein		1	1			mp	nucleotide		34	30		0	5	4	0
P16039	Nucleophosmin		1		1	ck,cp,ol,nu	cp,cob,mp,reg,	protein		27		42	0	2	0	3
P84229	histone H3		1		1			DNA		24		441	0	6	0	9
Q6EE58	Ribosomal protein S3			1	1	cp,rb	mp	RNA			23	77	0	0	1	5

Key. Cell components: ch chromosomal; ck cytoskeletal; cl cytosol; cp cytoplasm; cs cell surface; em endosome; er endoplasmic reticulum; gg golgi; nu nucleus; mb membrane; mt mitochondrial; ol organelle lumen; rb ribosome; sc splicosomal complex

Biological function Key: cc cell communication; ccm cellular component movement; cd cell division; cdf cell differentiation; ch cellular homeostasis; cob cell organisation and biosynthesis; cp cell proliferation; cx cell death; dfr defense response; mp metabolic process; rbp regulation of biological process; rep reproduction; res response to stimulus; tp transport

Grey indicates proteins with >1 identification with low Mascot (<50) and/or low peptide scoring
