# Supplementary material for: SILAC-iPAC: A quantitative method for distinguishing genuine from non-specific components of protein complexes by parallel affinity capture
Source: J Proteomics. 2015 Feb 6;115:143–56. doi: 10.1016/j.jprot.2014.12.006 (PMC4329988; doi:10.1016/j.jprot.2014.12.006)
Supplement: Supplemental Table S2 — Peptides identified using Mascot Percolator for (A) PI5P4K2β pull-downs and (B) FANCC pull-downs. (C) Known DNA repair proteins identified in FANCC pull-downs. [file mmc4.pdf]

Supplemental Table S2*A* . PI5P4K2 bait peptides observed in parallel purifications in replicate and reciprocal labelling experiments

Peptide	FLAG	TALON	reciprocals
**AAPGTVASVMASK**	1,2,3,4	3	✓
AAPGTVASVMASKTK		2	
AAPGTVASVMASKTKTK	2		
**ASDPLLSVLMWGVNHSINELSH VQIPVMLMPDDFK**	4		
**DNDFINDGQK**	3, 4	2, 3	
ELPTFKDNDFINDGQK	3		
ENMPSHFKFK	1		
**EVYFMAIIDILTHYDAK**	3		
**FGIDDQDFQNSLTR**	2, 3, 4	2, 3	✓
**FLDFIANILT**	2, 3, 4	2, 3	✓
**IHIDENNKR**	3		
**KEVYFMAIIDILTHYDAK**	3, 4	3	
KEVYFMAIIDILTHYDAKK	1, 2, 3	2	
KYDLKGSTVAR	3, 4	3	
**LMDYSLLVGIHDVER**	4	3	✓
**LTVDGVEIYMIVTR**	3		
RMFLEKLK	2		
SAPLANDSQARSGAR		4	
**TITSEDVAEMHNILK**	3, 4	1, 3	✓
YDLKGSTVAREASDK	1	3	
**AEQEEMEVEDR**	2, 3, 4		✓
**AVSSEDVAEMHNILK**	4		
**DNDFLNEGQK**	3, 4	2	✓
EVYFMAIIDILTPYDAKK	4		✓
EYCPLVFR	4		
**FFGPGEFDPSVDVYAMK**	4		✓
**FGIDDQDYQNSVTR**	2, 3, 4		✓
FKEYCPLVFR	4		
FLTTYDR	2, 3, 4		✓
FLTTYDRR	3		✓
GSTVSREASDK		3	
**IMDYSLLVGIHDVDR**	4		✓
**LTVDGVETYMVVTR**	2, 4		✓
NVFSHRLTVHR	4		
**SAPVNSDSQGR**	4		✓
YDLKGSTVSREASDK	3		
DVEFLAQLK	1, 2, 3, 4	3, 4	✓
EASDKEK		3	
EASDKEKAK	4		
HGAGAEISTVNPEQYSK	2, 3, 4	3, 4	✓
IKVDNHLFNK	2, 3, 4	4	✓
NVFSHR	3		✓
VDNHLFNK	2	1	✓

Key: α peptides (amber), β peptides (blue), peptides found in both (grey).
numbers indicate the experiment number in which the peptide was identified.
Bold indicated peptides used for **quantitation**
