# Supplementary material for: SILAC-iPAC: A quantitative method for distinguishing genuine from non-specific components of protein complexes by parallel affinity capture
Source: J Proteomics. 2015 Feb 6;115:143–56. doi: 10.1016/j.jprot.2014.12.006 (PMC4329988; doi:10.1016/j.jprot.2014.12.006)
Supplement: Supplemental Table S2 — Peptides identified using Mascot Percolator for (A) PI5P4K2β pull-downs and (B) FANCC pull-downs. (C) Known DNA repair proteins identified in FANCC pull-downs. [file mmc5.pdf]

Supplemental Table S2*B.* FANC peptides from Fanc core complex

Fanc Group	Protein Accession(s) (UniprotKB)	Peptide Sequence	CaM	IgG
A	B3Y9I3	AELDALR	1,2, 2r	1r,2,2r
A	B3Y9I3	AGAGGGTLR	1	
A	B3Y9I3, F1NLX0	CCCAVHPDSLQQFFR		1r
A	F1NLX0	EKMLQDKR		2
A	B3Y9I3	ELLQGRAR	2	
A	B3Y9I3, F1NLX0	KSTFGSTAELR	2r	
A	B3Y9I3, F1NLX0	LDLGSCGQLKSVSPDIHCK	1	
A	B3Y9I3, F1NLX0	LGVPTAVLAAK		1
A	B3Y9I3	LREYQSSAMGFLSSGR	2r	
A	B3Y9I3, F1NLX0	QARFQELLKEK	1	
**A**	**B3Y9I3, F1NLX1**	**total sequence coverage (%)**	**5.7**	**2.8**
B	B9A0U5	EQCMLSMHETMDGTLYQEVALK		1r
B	B9A0U5, E1BTB6	FMPHAYSR	1r	
B	B9A0U5, E1BTB6	KMKLDLR		2
B	B9A0U5, E1BTB6	LMKSGSK		1
B	B9A0U5	LQNEKEQCMLSMHETMDGTLYQEVALK	1	1,2,2r
B	B9A0U5, E1BTB6	MKKHNGVVEHLLLLLHSSDR	2r	
B	E1BTB6	MKPDQSSMEDIAAILAVSIRSSFQILSSDCTLTR	2r	
B	B9A0U5, E1BTB6	MLTGARFMPHAYSR		2
B	B9A0U5, E1BTB6	RVILQSCRALIDLVEGR		1r
B	B9A0U5, E1BTB6	SFLSSKESK		2r
B	E1BTB6	SPFEGVLTLFCR		1
B	B9A0U5, E1BTB6	SSFQIMSSDCTLTR	1	
B	B9A0U5, E1BTB6	SSGAFSVR	1	
B	B9A0U5, E1BTB6	VAEAQLSSDMIVWR	1r	
B	B9A0U5, E1BTB6	VVSSVCVCKSERLQK		2
**B**	**B9A0U5, E1BTB7**	**total sequence coverage (%)**	**14.5**	**15.0**
C	Q5F3H0, Q60GM0, F1NYH1	HNGGENQRTVK	2, 2r	2,2r
C	Q5F3H0, Q60GM0, F1NYH1	IVNEIFK		2r
C	Q5F3H0, Q60GM0, F1NYH1	MAQDTTVPK		2r
C	Q5F3H0, Q60GM0, F1NYH1	TQTMVESQAVYSRLMMLYNR		2r
**C**	**Q5F3H0, Q60GM0, F1NYH1**	**total sequence coverage (%)**	**1.9**	**8.4**
E	F1NHZ9	CLCQKIAQSK		2
E	F1NHZ9	IAQSKAEGRR	2r	
E	F1NHZ9	VLEESLEFSEEREGK	2	
**E**	**F1NHZ9**	**total sequence coverage (%)**	**3.0**	**2.0**
F	E1C4T8	AFAVVAEALLR	2r	
F	E1C4T8	FRSRPQLRDALR		2
F	E1C4T8	LAPLARRK		1r
**F**	**E1C4T8**	**total sequence coverage (%)**	**2.8**	**5.1**
G	Q7SZH6, Q5ZHU1, F1P3G7	EAIMQFSR		2
G	Q7SZH6, Q5ZHU1, F1P3G7	LRCGTAPEPGCLQAWAAECEALAGR		1
**G**	**Q7SZH6, Q5ZHU1, F1P3G7**	**total sequence coverage (%)**	**0**	**5.1**
L	Q3MUH5	DLLEIDFPSR		1r
**L**	**Q3MUH5**	**total sequence coverage (%)**	**0**	**2.7**
M	F1NE03	ADVQLTANRK	1	
M	F1NE03	DVPSLTK	1	
M	F1NE03	EFIYSHPKLK	2	
M	F1NE03	ELALLEQRK	2r	2r
M	F1NE03	GNDASSFMER	1	
M	F1NE03	IIVLSDSSGEETNVSNEK		1r
M	F1NE03	KTDQTTSGGTCVDTR		1r,2r
M	F1NE03	LVQRMGRTGR		2r
M	F1NE03	RDVPSLTK	1r	1
M	F1NE03	RGNDASSFMERTCKPALHLQNPNR		2r
M	F1NE03	RNKGHLSTTSSAFAQK	1	
M	F1NE03	SPIRLVQRMGR		2r
M	F1NE03	VNPNSQR	1	
M	F1NE03	VQVCSLGSGDYVVSNRMAVERK	1r	
M	F1NE03	YKPNDSGRLPK		1
**M**	**F1NE03**	**total sequence coverage (%)**	**6.2**	**3.8**
FAAP100	A5A748, R4GG85	LMTCGLCSPEDADVELTPAEAGRRIK		1r
FAAP100	A5A748, R4GG85	MVAVKADQRDETTVPELR		2r
**FAAP100**	**A5A748, R4GG85**	**total sequence coverage (%)**	**0**	**4.9**

Key: 1,2 replicates 1 and 2; r= reciprocal labelled experiment.
CBP=calmodulin binding protein tag Calmodulin pulldown, IgG=IgG pulldown of Protein A tag
