# Supplementary material for: SILAC-iPAC: A quantitative method for distinguishing genuine from non-specific components of protein complexes by parallel affinity capture
Source: J Proteomics. 2015 Feb 6;115:143–56. doi: 10.1016/j.jprot.2014.12.006 (PMC4329988; doi:10.1016/j.jprot.2014.12.006)
Supplement: Supplemental Table S2 — Peptides identified using Mascot Percolator for (A) PI5P4K2β pull-downs and (B) FANCC pull-downs. (C) Known DNA repair proteins identified in FANCC pull-downs. [file mmc6.pdf]

Supplemental Table S2*C* . Fanconi interacting proteins

Genes	Uniprot IDs	Description	Calmodulin Pulldown rep 1	Calmodulin Pulldown rep 2	Calmodulin Pulldown r'cal 1	Calmodulin Pulldown r'cal 2	IgG Pulldown rep 1	IgG Pulldown rep 2	IgG Pulldown r'cal 1	IgG Pulldown r'cal 2
FANCD2	Q68Y81, FINP22	Fanconi anemia protein FANCD2	1	1	0	3	3	1	1	2
FANCI	B0I564, F1P1Z1	Fanconi anemia complementation group I	1	2	0	1	3	1	2	2
FANCJ/BRIP1	Q3YK19	Fanconi anemia group J protein homolog, helicase	1	3	0	0	3	0	3	0
BRCA1	F1N8I5, Q90Z51	Breast and ovarian cancer susceptibility-like protein	1	2	1	2	7	4	4	5
FAM175 (ABRAXAS)	Q5ZHS0, E1C3L2	BRCA1-A complex subunit Abraxas	0	1	0	1	3	1	0	0
BRE	Q5ZML0	BRCA1-A complex subunit BRE	0	1	0	0	0	2	0	0
FANCD1/BRCA2	Q8QFV6, F1P3B2	Breast cancer type 2 susceptibility protein	3	6	3	4	4	9	11	6
PALB2/FANCN	F1NAU2	Partner and localizer of BRCA2	0	1	0	0	1	0	1	0
RAD51	P37383	DNA repair protein RAD51 homolog 1	1	0	0	2	1	2	3	1
FAN1	F1NC90	Fanconi-associated nuclease 1	0	0	2	1	0	2	3	1
SLX4/FANCP	F1NKN4	Structure-specific endonuclease subunit SLX4	1	0	0	1	1	2	4	1
BLM	Q9I920, D3KR64, F1P3V1	Bloom syndrome protein homolog/BLM helicase	0	5	5	2	2	1	4	2
TOPBP1	E1BSJ5	DNA topoisomerase 2-binding protein 1	0	1	0	1	1	2	0	0
RMI1	Q5ZHV8, F1P2E5	RecQ-mediated genome instability protein 1	x	x	x	x	x	x	x	x
RMI2	Q5ZM20	RecQ-mediated genome instability protein 2	2	0	1	0	1	0	1	0
ATM	E1C0Q6, Q9PVT8	Serine-protein kinase ATM	4	2	6	6	9	10	7	10
ATR/Gga.21455	F1NGW1	Serine/threonine-protein kinase ATR	5	3	3	6	6	4	7	3
ATRIP	F1NGS1	ATR-interacting protein	0	1	2	1	0	0	3	0
**CHEK1 (CHK1)**	Q8AYC9	Serine/threonine-protein kinase Chk1	1	2	1	4	2	3	1	7
CHEK2 (CHK2)	A1EAT1, F1N864	Checkpoint and tumor suppressor protein 2	2	1	0	1	0	1	0	0
ERCC4/XPF/FANCQ	F1NAV2	DNA repair endonuclease XPF	0	2	2	2	3	3	1	3
EME1	F1NU84	Crossover junction endonuclease EME1	1	0	1	0	1	0	0	1
**CDK5**	B6E1W1	Cyclin-dependent kinase 5	1	2	0	4	1	3	2	2
RAD18	F1NVL6, Q5ZIP0	Postreplication repair E3 ubiquitin-protein ligase	2	1	2	0	1	0	2	1
UBE2T	F1NM75	Ubiquitin-conjugating enzyme E2 T	0	0	0	0	1	0	1	0
UBE2W	F1NEJ6	Ubiquitin-conjugating enzyme E2 W	0	0	0	1	0	0	0	1
PRKDC (DNA-PK)	Q8QGX4, F1NQD1, Q6S5B0	DNA-dependent protein kinase catalytic subunit	4	13	6	9	3	10	11	10

Key: rep =replicates, r'cal=reciprocals, **bold** are quantified proteins. Known repair proteins (dark grey), regulatory proteins (light grey), checkpoint proteins (white)
