# Supplementary material for: SILAC-iPAC: A quantitative method for distinguishing genuine from non-specific components of protein complexes by parallel affinity capture
Source: J Proteomics. 2015 Feb 6;115:143–56. doi: 10.1016/j.jprot.2014.12.006 (PMC4329988; doi:10.1016/j.jprot.2014.12.006)
Supplement: Supplemental Table S4 — FANCC associated proteins that had at least one statistically significant protein from replicate datasets that were pulled down with both calmodulin and IgG pull-downs (A), calmodulin resins (B) or IgG resins only (C). [file mmc8.pdf]

Supplemental Table S4*A* . Proteins with ≥1 statistically significant ratio identified in both CBP and IgG pulldowns .

				**Calmodulin pulldown**				**IgG pulldown**			
**UniprotKB IDs**	**Gene**	**Description**	**location**	**FHDL C1**	**FHDL C2**	**DHFL C1**	**DHFL C2**	**FHDL I1**	**FHDL I2**	**DHFL I1**	**DHFL I2**
P16039;Q6LEK3	NPM1	Nucleophosmin	nuc	**2.490**	**4.034**	1.653	1.973	**7.377**	1.032	0.950	1.005
E1C540	NPM3	Nucleoplasmin-3 I chaperone	nuc	**2.902**	**2.253**	1.543	0.765	**6.611**	**1.697**	1.067	0.696
F1N8Z4;Q5ZIC4	RUVBL1	DNA repair. ATP- dependent DNA helicase (3' to 5')	nuc		**1.951**		0.505		**3.026**		**0.233**
Q5ZMN1;E1C769	G3BP1	DNA-unwinding enzymes	nuc	0.818	**1.927**		0.376	1.355	**2.151**	1.727	**0.358**
Q5XNV3	CAPRIN1	cell cycle associated protein	PM	0.793	**2.383**		0.359	**1.997**	**7.010**		**0.251**
F1P5X5;Q5F3B6	RCC2	telophase disk protein	nuc		**2.167**		0.831		**1.915**		
F1NJS6;Q5ZMA9	KPNA2	Importin subunit	nuc env		**4.429**		**0.208**	0.787	**2.908**		**0.216**
Q5ZI10	WBSCR22	DNA methylation	nuc		**2.065**		0.533		**2.560**		**0.515**
F1P5K0;Q5F456;F1	PTBP1	ribonucleoproteins (hnRNPs). RNA-binding	nuc		**2.909**		0.603		**1.840**		
Q5ZIH1	HNRNPD	nucleic acid binding proteins complex with heterogene	nuc	0.927	**1.843**	1.749	0.879	**2.891**	**2.416**		0.930
Q5F3I2; E1BQF9	CSDE1	cold shock domain containing E1, RNA-binding	cyt		**2.372**		0.522		**2.192**		**0.318**
Q45KQ2;Q5ZLU8	RCJMB04_4m1	Aggrecan promoter binding protein	nuc	**1.552**		1.202	0.539	**2.371**	**1.567**		**0.512**
Q5F491;F1NIX2; A7VJB0;Q9DGR8;	RCJMB04_2a4; DDX3X; CPL10;	Dead Box helicase	nuc	0.980	**2.287**	1.240	0.319	**2.675**		0.548	
P0C1H5;F1NF30	H2B-VII	Histone 2B7	nuc		**1.811**	0.700	1.205	0.834	**1.355**	1.430	1.049
Q9DEQ6; E1BT82	EIF2S2;EIF2B	Eukaryote initiation factor 2	cyt	0.846	**2.478**		0.549	0.893	**1.856**		0.769
P18660	RPLP1	acidic ribosomal protein P1	cyt		**3.761**		1.036		**2.335**		
E1BU66	RPL38	ribosomal	cyt		**2.201**			1.065	**1.326**		
E1BUZ5	RPLP2	60S acidic ribosomal protein P2	cyt		**2.566**		1.191	0.799	**2.007**		
F1NH93	RPS20	ribosomal	cyt	1.017	**2.547**	2.072	0.367	1.395	1.490	0.829	**0.422**
F1P304	ATP5O	ATP synthase subunit O	mit		**2.771**		0.378	**2.888**	**1.935**	0.827	**0.350**
E1C658	ATP5H	ATP synthase subunit d	mit		**3.461**		0.278	**2.970**	**1.578**	1.301	**0.313**
F1NSC1	ATP5F1	ATP synthase subunit b	mit		**2.236**		0.348	**2.101**	**1.590**		**0.398**
F1NZ24; Q5ZLZ0	SLC25A3	Phosphate carrier protein	mit	**3.755**	0.761	0.692	0.526	**2.083**	0.715	0.760	0.764
F1NNH9	TOMM70A	Mitochondrial import receptor subunit	mit		**1.902**		0.338		**2.827**		**0.451**
F1NBW0	MTPAP	mitochondrial poly(A) polymerase	nuc mit		**2.039**	0.364			**1.348**		0.626
E1C5I9	SRPK1	Serine/threonine-protein kinase splicing factor	nuc, cyt		**3.685**		0.234		**4.567**		**0.152**
F1NBN0	MTDH	oncogene LYRIC	ER		**5.371**		0.245		**4.205**		**0.194**
P01875	IGHM	Ig mu chain c	ER	0.164	0.733	**0.209**	1.654		0.748	**0.183**	0.793
F1NV93;P24367	PPIB	pp isomerase	ER	**3.142**	**2.635**	0.723	0.245	**5.618**	**2.920**	0.437	**0.342**
F1NK96;Q5F472	PDIA6	Part of a large chaperone multiprotein complex	ER	**2.166**	1.529	0.750	0.958	**3.028**	1.127	0.534	1.088
F1NW97;P09644	TBA5	Tubulin alpha 5 chain	cyt		**12.380**		5.246		**1.594**		1.506
			median	***0.815***	***0.649***	***1.120***	***1.357***	***0.984***	***0.621***	***0.981***	***1.712***
			med +/- 1sd	***1.508***	***1.735***	***0.337***	***0.218***	***1.872***	***1.310***	***0.220***	***0.560***

Key: **Bold** indicates ratio +/- 1SD of median. Heatmap annotation identifies enriched high confidence ratios (green) decreasing to low confidence or contradictory ratios (red).

Supplemental Table S4*B*. Proteins with ≥1 statistically significant ratio identified in Calmodulin pulldowns only.

UniprotKB IDs	Gene name	Description	location	FHDL_C	FHDL_C	DHFL_C	DHFL_C	No. sig.
Q5F489;F1NZW0	F1NZW0	Transcription initiation factor TFIID subunit	nuc	**3.113**	**6.189**	0.490	**0.103**	3
Q5ZKC3	RCJMB04_11n1	uncharacterised, SAM/SH3 domain-containing		**3.051**	**15.490**	1.329	**0.118**	3
F1NCD4	MYH9	Myosin 9	cyt	**2.334**	**2.228**	0.309	**0.687**	3
Q90753	brg1/SMARCA4	regulation of transcription, DNA-dependent	nuc		**3.084**	**0.155**	**0.136**	3
E1C2F9	IQGAP2	small GTPase mediated signal transduction	cyt		**6.945**		**0.182**	2
Q5ZLM0	CDC73	Parafibromin, Cell division cycle protein 73 homolog,	nuc		**3.775**		**0.207**	2
F1P0U1	LOC101749184	E3 ubiquitin-protein ligase UBR5	cyt		**3.406**		**0.123**	2
F1N6T4	FECH	Ferrochelatase, mitochondrial	mit		**2.133**		**0.200**	2
F1P5V4	HELLS	methylation-dependent chromatin silencing,	nuc		**4.744**	0.947	**0.126**	2
E1BTZ4	TMEM214	Transmembrane protein 214	PM		**11.084**	2.290	**0.157**	2
P02607	MYL6	Myosin light polypeptide 6	cyt		**4.336**		0.219	1
E1C555	SMARCD2	SWI/SNF-related matrix-associated actin-dependent	nuc		**3.830**		0.304	1
F1NY82	IQGAP1	Ras GTPase-activating-like protein IQGAP1	nuc cyt		**3.732**		0.219	1
F1NF84	GOLGA2	Golgin subfamily A member 2	golgi		**2.924**		0.283	1
F1P1A8	SMARCC1	SWI/SNF-related matrix-associated actin-dependent	nuc		**2.858**		0.401	1
G5H7N8;F1NGV1	ZC3H15	Zinc finger CCCH domain-containing protein 15 (DRG	nuc cyt		**2.790**		0.504	1
F1ND93	PTCD3	Pentatricopeptide repeat domain-containing protein	mit		**2.215**		0.435	1
A5HUJ1	KIFC1	Carboxy-terminal kinesin 1	cyt		**2.214**		0.497	1
E1C524	RALA	Uncharacterised, actin cytoskeleton reorganization,	cyt PM		**2.201**		0.330	1
B6V3H7	CAPN1	Mu-calpain large subunit, Calcium-activated neutral	cyt		**2.199**		0.423	1
F1N8T0	NEMF	Nuclear export mediator factor	nuc		**2.189**		0.338	1
Q765Y9;E1BX55	DECR1	2,4-dienoyl-CoA reductase	nuc mit		**2.147**		0.325	1
Q5ZJ83	RCJMB04_20c5	uncharacterised, protein transporter activity	PM		**2.093**		0.347	1
E1C1S5	SUPT6H	regulation of DNA-dependent transcription,	nuc		1.313		**0.183**	1
F1NR99	CPOX	Coproporphyrinogen-III oxidase	mit		1.694		**0.188**	1
F1NGU3;F1NX33	LRPPRC	Leucine-rich PPR motif-containing protein	nuc mit	**2.236**	1.440	0.979	0.553	1
Q5ZLG7	RCJMB04_6e4	transmembrane transport	mit	**1.810**	1.299	0.898	0.399	1

Key: **Bold** indicate ratios +/- 1SD of median. Heatmap annotation identifies enriched high confidence ratios (green) decreasing to low confidence or contradictory ratios (red).

Supplemental Table S4*C* . Proteins with ≥1 statistically significant ratio identified in IgG pulldowns only.

UniprotKB IDs	Gene name	Description	location	FHDL_I1	FHDL_I2	DHFL_I1	DHFL_I2	No. sig.
P84172	TUFM	Elongation factor Tu	mit	1.030	**1.311**	0.655	**0.521**	2
F1NJLC	ATXN2	ATXN2 protein (Ataxin-2)	nuc cyt golgi	**2.527**	1.214		**0.447**	2
042395; 057348	CNBP	Cellular nucleic acid-binding protein (Zinc finger	nuc cyt ER		**H**		**0.103**	2
Q52JB0	DDX47	Probable ATP-dependent RNA helicase DDX47 (DEAD	nuc		**1.639**		**0.451**	2
Q5H7M6;F1NXI3	DRG1	Developmentally-regulated GTP-binding protein 1	nuc cyt		**2.494**		**0.409**	2
F1P3YI	EXOSC1	Exosome complex component CSL4 (Exosome	nuc		**1.400**		**0.394**	2
E1C525	EXOSC10	Exosome component 10	nuc		**1.749**		**0.283**	2
F1N591	RPL9	Ribosomal_L6	cyt		**1.317**		**0.510**	2
F1NBM0	OGFOD1	2-oxoglutarate and iron-dependent oxygenase	cyt		**1.663**		**0.556**	2
F1NWH1;RG5150	RPAP3	RNA polymerase II-associated protein 3	cyt		**2.291**		**0.290**	2
E1BVX9;Q5ZK25	SRP72	Signal recognition particle subunit SRP72	cyt		**1.553**		**0.511**	2
Q04678;B62LK1	SSRP1	Recombination signal sequence recognition protein 1	nuc		**1.711**		**0.473**	2
O42283	HSP10	10 kDa heat shock protein	mit	**3.334**		0.454		1
F1NPL2	PCBP3	Poly(rC)-binding protein 3	cyt	**2.108**	1.271		0.644	1
P35458	DCTN1	Dynactin subunit 1	cyt		1.166		**0.547**	1
Q5ZKC1;Q52LX2	EIF2A	Eukaryotic translation initiation factor 2A	cyt		**1.426**		**0.780**	1
F1NCI5	HNRNPA0	Heterogeneous nuclear ribonucleoprotein A0	nuc		1.118		**0.524**	1
F1NDA0	NMT1	Glycylpeptide N-tetradecanoyltransferase 1	cyt PM		1.679		0.581	1
E1C080	RAB8B	Ras-related protein Rab-8B	peroxisome me	**2.449**		0.788		1
F1NSA8	RAP1A	Ras-related protein Rap-1A	cyt PM		**1.752**	0.457		1
Q5ZJJ2	RPA1	Replication protein A 70 kDa DNA-binding subunit	nuc		**1.864**		0.670	1
E1C2C3	SF3B1	Splicing factor 3B subunit 1	nuc	1.399	1.277	1.065	**0.497**	1
Q5F422	DEK	regulation of double-strand break repair via	nuc	1.658	**2.065**			1
E1BSR5	RBM25	RNA-binding protein 25	nuc cyt	1.515	**1.532**			1
F1NZU2	SYNCRIP	Synaptotagmin-binding, cytoplasmic RNA-interacting	nuc cyt		**1.340**		0.777	1
Q5ZJL7	WDR76	DNA damage-binding protein 2	nuc		**1.995**		0.821	1
E1BRW7	TTC35	Tetratricopeptide repeat protein 35	mit ER	**2.506**	**3.088**		1.702	2

Key: **Bold** indicate ratios +/- 1SD of median. Heatmap annotation identifies enriched high confidence ratios (green) decreasing to low confidence or contradictory ratios (red). H denotes protein found in heavy channel only.
